# Snowbeds are more affected than other subalpine–alpine plant communities by climate change in the Swiss Alps

**DOI:** 10.1002/ece3.2354

**Published:** 2016-09-09

**Authors:** Magalì Matteodo, Klaus Ammann, Eric Pascal Verrecchia, Pascal Vittoz

**Affiliations:** ^1^ Institute of Earth Surface Dynamics (IDYST) University of Lausanne Géopolis Building 1015 Lausanne Switzerland; ^2^ Prof. Emeritus University of Bern Monruz 20 2000 Neuchâtel Switzerland

**Keywords:** Colonization, cover changes, diversity, ecological indicator values, grasslands, homogenization, resurvey study, semipermanent plot, snowmelt, Switzerland

## Abstract

While the upward shift of plant species has been observed on many alpine and nival summits, the reaction of the subalpine and lower alpine plant communities to the current warming and lower snow precipitation has been little investigated so far. To this aim, 63 old, exhaustive plant inventories, distributed along a subalpine–alpine elevation gradient of the Swiss Alps and covering different plant community types (acidic and calcareous grasslands; windy ridges; snowbeds), were revisited after 25–50 years. Old and recent inventories were compared in terms of species diversity with Simpson diversity and Bray–Curtis dissimilarity indices, and in terms of community composition with principal component analysis. Changes in ecological conditions were inferred from the ecological indicator values. The alpha‐diversity increased in every plant community, likely because of the arrival of new species. As observed on mountain summits, the new species led to a homogenization of community compositions. The grasslands were quite stable in terms of species composition, whatever the bedrock type. Indeed, the newly arrived species were part of the typical species pool of the colonized community. In contrast, snowbed communities showed pronounced vegetation changes and a clear shift toward dryer conditions and shorter snow cover, evidenced by their colonization by species from surrounding grasslands. Longer growing seasons allow alpine grassland species, which are taller and hence more competitive, to colonize the snowbeds. This study showed that subalpine–alpine plant communities reacted differently to the ongoing climate changes. Lower snow/rain ratio and longer growing seasons seem to have a higher impact than warming, at least on plant communities dependent on long snow cover. Consequently, they are the most vulnerable to climate change and their persistence in the near future is seriously threatened. Subalpine and alpine grasslands are more stable, and, until now, they do not seem to be affected by a warmer climate.

## Introduction

During the end of the 20th century (1975–2004), the mean annual temperature in Switzerland increased by 0.57°C per decade with a stronger trend in spring and summer seasons (Rebetez and Reinhard 2008). After a gradual increase until the early 1980s, snow precipitation in Switzerland significantly decreased (Laternser and Schneebeli 2003) with a particularly pronounced trend at lower elevations (501–800 m a.s.l., Serquet et al. 2013). Snowfall decreased above 1700 m as well, but only at the beginning and at the end of the winter season (Serquet et al. 2013). At such elevations, winter temperatures are generally much lower than the melting point, and, even with warmer conditions, there is little potential for a decrease in snowfall days (Serquet et al. 2011). By contrast, the combination of higher temperatures and lower snowfalls during the spring season results in a lower snow cover (IPCC, 2014), earlier melt‐out dates, and longer growing seasons for plants (Dye 2002). Future scenarios predict the continuation of this trend through the 21st century and indicate that vegetation of high latitudes and elevations is the most threatened (ACIA, 2005; IPCC, 2014).

Impacts of the recent climate change on alpine vegetation have been largely recorded by many long‐term studies on European upper alpine and nival summits. Authors observed an increase in species richness during the last century (see Stöckli et al. 2011 for a review), already noticeable on a shorter timescale (2001–2008; Pauli et al. 2012). The newly arrived species are subalpine and lower alpine species (Vittoz et al. 2008a; Engler et al. 2011) and now, because of longer growing seasons, they are able to grow at higher elevations. Space on the summits is not a constraint to colonization as it is widely available. However, the upward shift of plant species led not only to higher species number, but also to a homogenization of plant composition across Alpine Swiss summits (Jurasinski and Kreyling 2007). Similarly, vegetation of the high northern latitudes has been changing over the past few decades and a general increase in biomass and proliferation of shrub species are responsible for the tundra “greening” (see Epstein et al. 2013 for a review).

Many more uncertainties exist about the effects of climate warming at lower elevations. A shift of tree line northwards and to higher elevations is the most often observed change on European mountain ranges (see Garamvoelgyi and Hufnagel 2013 for a review). In the Swiss Alps, the forest limit moved upward with a mean decadal increment of 28 m between 1985 and 1997 (Gehrig‐Fasel et al. 2007). However, between tree line and the upper alpine–nival belt, there is a wide range of plant communities whose responses to altered temperatures and precipitations have been poorly investigated so far. This is unfortunate, as identifying the most threatened plant communities is very important to establish proper conservation measures. Some previous long‐term surveys focused on changes of specific plant community, such as alpine siliceous grasslands (Dupré et al. 2010; Windmaißer and Reisch 2013), calcareous grasslands (Kudernatsch et al. 2005; Vittoz et al. 2009), or snowbed communities (Carbognani et al. 2014; Pickering et al. 2014; Sandvik and Odland 2014). However, only a couple of studies located in the Scottish highlands (Britton et al. 2009; Ross et al. 2012) and one in the Italian Alps (Cannone and Pignatti 2014) looked at long‐term vegetation changes in a variety of alpine plant communities.

At these elevations, the effects of climate and land‐use changes are difficult to disentangle. Indeed, seasonal grazing has been decreasing and many pastures have been abandoned since the end of the nineteenth century (Bätzing 1991). This highly contributed to the forest expansion toward higher elevations (Gehrig‐Fasel et al. 2007; Vittoz et al. 2008b) and favored the arrival of plants from fallow and wood edge communities in the subalpine grasslands (Vittoz et al. 2009). Moreover, as a result of industrial, traffic, and agronomic emissions, tropospheric concentrations of nitrogen compounds have increased remarkably, reaching levels that are likely to affect the aboveground productivity of alpine plants (Bassin et al. 2007).

It has been demonstrated that nitrogen deposition causes a decrease in species richness in the Swiss montane grasslands, with oligotrophic, and usually rare, species being particularly disfavored (Roth et al. 2013). Subalpine and alpine grasslands are likely more vulnerable to negative effects of N deposition, as they have shorter growing seasons and generally thinner and nutrient poorer soils (Bowman et al. 2012). However, increased N depositions may have different consequences between habitats: using a plant trait analysis, Maskell et al. (2010) showed that eutrophication and acidification occurred, both of which can be responsible for species loss. Indeed, in a moss‐dominated alpine heath of Northern Europe, N deposition seems to trigger a decline of plant diversity and of shrub, bryophyte and lichen covers, but an increase in the graminoid cover (Armitage et al. 2014).

A powerful and widely used tool to identify factors driving the vegetation changes is the species indicator values of Landolt et al. (2010) for the flora in the Alps or those of Ellenberg et al. (1991) in Central Europe. These semiquantitative parameters, although inferred from field experience and not from direct measurements, have been shown to give pertinent indications of the species ecological optima within small spatial areas in Alpine landscapes (Scherrer and Körner 2011). Specifically, the temperature indicator value is significantly correlated with the average soil temperature, which is far more representative of actual conditions experienced by low‐stature alpine plants than the air temperature interpolated from meteorological stations (Scherrer and Körner 2011).

For the purpose of this study, 63 exhaustive plant inventories performed on six plant community types during the period 1964–1990 and located between the subalpine and alpine belts of the Swiss Alps have been revisited. Through a time comparison of species frequencies and cover, and with the help of indicator values, the following questions are targeted: (1) Are there observable changes in the subalpine–alpine vegetation over the last 25–50 years in species richness and community composition in the Alps? (2) Do the magnitude and direction of changes vary across different plant communities and how? (3) What are the environmental conditions that can explain the observed changes?

## Materials and Methods

### Study sites

**Figure 1:**
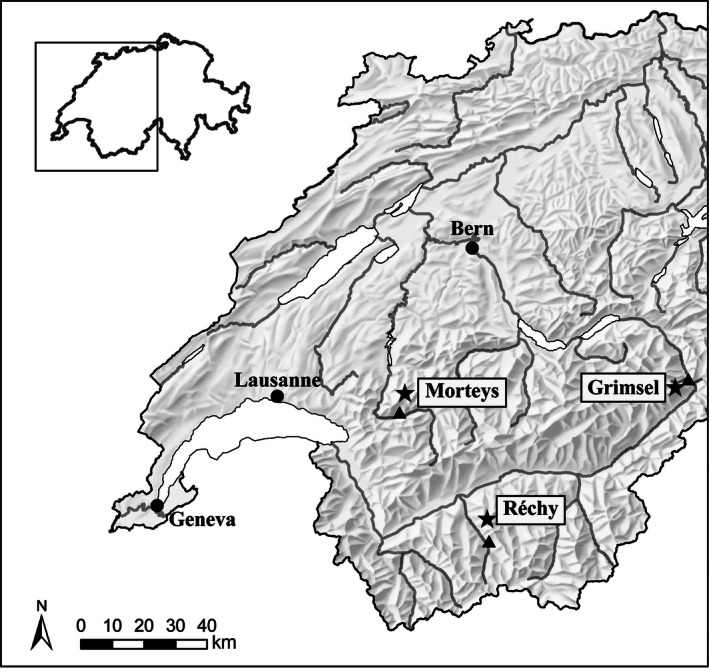
Study site area. Stars represent the three study sites, and triangles, the corresponding meteorological stations (Château‐d'Oex for Morteys, Grimsel Hospiz for Grimsel, Evolène for Réchy).

**Figure 2:**
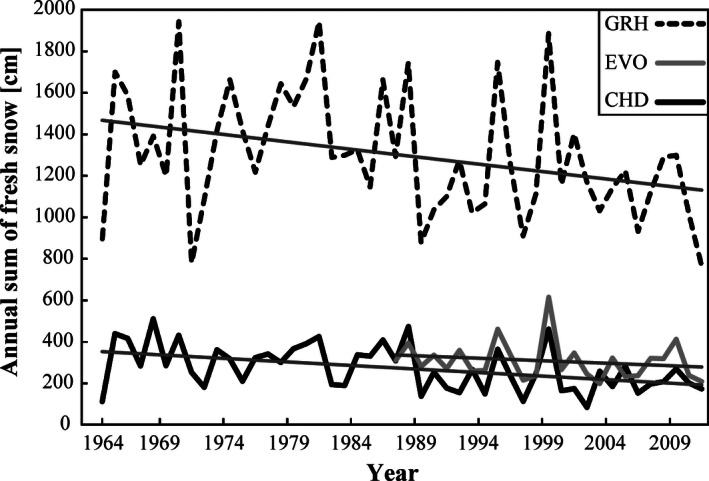
Annual sum of the fresh snow thicknesses daily measured at 5:40 a.m. from 1964 to 2011 (at Château‐d'Oex – CHD, and Grimsel Hospiz – GRH weather stations) and from 1987 to 2011 at the Evolène (EVO) weather station (MeteoSwiss network, Begert et al. 2005). The overall decrease in the snow amount among the three stations is significant (ANCOVA test, *P*‐value < 0.001).

Three study sites are located in the Northern Alps and western central Alps of Switzerland (Fig. 1). The Northern Alps are characterized by higher precipitations than the Central Alps. The Morteys area (46°32′N, 7°09′E) is situated on a calcareous bedrock with karstic geomorphology. The plots are located between 1698 and 2232 m a.s.l., in the transition from the subalpine to the lower alpine belt. The mean annual temperature is about 2.1°C, and the annual precipitations are 1650 mm (Zimmermann and Kienast 1999). The annual sum of fresh snow thickness decreased by 34.1 cm per decade between 1964 and 2011, while the mean summer temperature (from June to September) increased by 0.47°C per decade during the same period at the closest meteorological station (Château‐d'Oex, 1029 m; Fig. 2 and Appendix S1).

The Grimsel area (46°32′N, 8°16′E) is situated on gneiss and granodiorite bedrocks (Oberhänsli et al. 1988). The slopes in the Grimsel Valley are covered by various moraine deposits from the last maximum glacier advances that occurred between 1860 and 1920 (Ammann 1979). The plots are situated in the lower alpine belt, between 2310 and 2650 m a.s.l., and are characterized by mean annual temperature and precipitations of −0.44°C and 2071 mm, respectively (Zimmermann and Kienast 1999). The annual sum of fresh snow thickness decreased in average by 71.2 cm per decade, and the mean summer temperature rose by 0.41°C per decade between 1964 and 2011 (Grimsel Hospiz, 1980 m; Fig. 2 and Appendix S1).

The Réchy area (46°10′N, 7°30′E) is located on a mixed bedrock composed by gneiss, mica schists, quartzite, calcshists, marble, and cornieule and is shaped by geomorphological processes related to glaciers, gravity movements, and cryoturbation. A mosaic of acid and alkaline soils characterizes the area. Elevation of the vegetation plots ranges from 2328 to 2697 m a.s.l., namely the tree line ecotone and the lower alpine belt of the region. The area is the coldest and the driest among the three study sites, with a mean annual temperature of −0.53°C and 1480 mm of annual precipitations (Zimmermann and Kienast 1999). The annual sum of fresh snow thickness decreased by 24.1 cm, whereas the mean summer temperatures increased by +0.25°C per decade (Evolène, 1825 m; Fig. 2 and Appendix S1) during the 1987–2013 time span (no data available before).

The three study sites have been partially included in natural reserves for several decades. Except for Grimsel, where there has been no cattle grazing since 1953, the two other sites are currently pastured in some parts. Thanks to the natural reserve management in Morteys, the land use (cow and goat grazing) has barely changed during the last 40 years. In Réchy, the type and amount of cattle have fluctuated since the 1970s with alternating cow and sheep grazing, proportions depending on both elevation and location.

The total nitrogen deposition in Morteys and Grimsel areas for the year 2007 amounted on average to 10.4 and 6.8 kg N·ha^−1^·year^−1^, respectively (according to Roth et al. 2013; data from FOEN Federal Office for the Environment). Data for the Réchy area were not calculated, but are comparable to those of Grimsel area because of the similar elevations and distance to main towns.

### Vegetation data

In order to have a complete overview of reactions of subalpine–lower alpine vegetation to climate change, six common vegetation types, for which more historical data are available, were selected (Table 1 and Appendix S2). Each vegetation type corresponds to a phytosociological alliance given between brackets: calcareous grasslands (*Seslerion*) located in the subalpine–alpine belt, generally on very steep, south‐exposed slopes; windy ridges (*Elynion*) in alpine belt, situated mostly on calcareous substrates; siliceous subalpine grasslands (*Nardion*); siliceous alpine grasslands (*Caricion curvulae*); typical snowbeds (*Salicion herbaceae*) associated with very long snow cover and acidic soil conditions; wet snowbeds (*Caricion bicolori‐atrofuscae*) also associated with very long snow cover, but close to running water, brought by rivers or firn melting, or close to lakes.

**Table 1:** Number of plots, time spans, authors, and elevation ranges of historical and recent surveys ordered by study site (upper part) and plant community (lower part). The names of the historical botanists are abbreviated as follows: Jean‐Louis Richard (JLR), Klaus Ammann (KA), Benoît Bressoud (BB), Olivier Duckert (OD). Numbers in brackets refer to medians

Site	No. of plots	Historical survey	Author(s) of historical data	Elevation (m)
Morteys	12	1972–1979 (1973)	JLR	1698–2232 (1884)
Grimsel	25	1964–1973 (1970)	KA	2310–2650 (2329)
Réchy	26	1977–1990 (1981)	BB, JLR, OD	2328–2697 (2567)
Plant community				
Calcareous grasslands	10	1972–1973 (1973)	JLR	1698–2099 (1807)
Windy ridges	13	1975–1990 (1979)	BB, JLR, OD	2180–2697 (2430)
Siliceous subalpine grasslands	12	1964–1973 (1967)	KA	2312–2370 (2320)
Siliceous alpine grasslands	11	1965–1989 (1970)	JLR, KA	2300–2682 (2528)
Typical snowbeds	8	1970–1981 (1973)	BB, JLR, KA	2313–2685 (2460)
Wet snowbeds	9	1977–1990 (1988)	JLR	2468–2677 (2585)

Among the available data, a selection of the most promising historical records was performed according to criteria of reliability and possibility to relocate them. The historical records were achieved by several botanists from 1965 to 1990 (Table 1) with most data being collected during the 1970s (1980s in the case of wet snowbeds). The inventories were only partly published (Ammann 1974; Richard et al. 1977, 1993), but field books were available for most of them and they represented the main information source. Because of their localization on topographic or vegetation maps (1:25,000 or more precise), the plot areas were approximately localized in the field, with a precision of ± 10–50 m. Each area was extensively visited, and, on the basis of information contained in the historical field books (site description, elevation, surface, slope, and exposition), the possible plot sites were defined. The exact plot location was selected in order to have a species composition as close as possible to the historical one. This permits a conservative approach of potential changes. When no area corresponded to the historical description, or when vegetation was markedly different, the site was discarded. Only historical records separated by a distance >10 m were retained in order to avoid spatial autocorrelation. Finally, 63 plots have been localized with a high confidence level. A new exhaustive record of all vascular plants was performed during summers 2013 or 2014 at the phenological optimum, within the same area as the historical one. Species cover was visually estimated, as in historical inventories, according to cover classes of Braun‐Blanquet (1964; Table 2). The plots were marked with metal plates in soil and the four corners measured with a high precision GPS (GeoXT, Trimble, Sunnyvale, CA) in order to enable their future use as permanent plots. Finally, the nomenclature of species is according to Aeschimann et al. (1996).

**Table 2:** Braun‐Blanquet's scale used in both historical and recent inventories to estimate plant cover, the corresponding cover range and medians, used in analyses of cover changes. Numerical codes used in all other analyses are also listed

Braun‐Blanquet's code	Cover range	Median of the cover range (%)	Numerical code (Gillet 2000)
*r*	1 or 2 individuals	0.05	0.1
+	<1%	0.5	0.5
1	1–5%	3	1
2	6–25%	15	2
3	26–50%	37.5	3
4	51–75%	62.5	4
5	76–100%	87.5	5

### Data analyses

The potential mistakes in species identifications, or changes in nomenclature and aggregation level between the two periods, were corrected by a scrupulous check of possible synonymies and by aggregating the pairs of species with frequent confusions into the same taxon. One frequent problem in plant monitoring studies is the overlooked species in one of the surveys (Vittoz and Guisan 2007; Burg et al. 2015). This bias is particularly likely to cause artifact in this study, as recent inventories involved generally two botanists instead of one in the historical records, and because the historical inventories, especially those of Richard et al. (1977), were not performed for monitoring purposes, but for the classification of plant communities. Changes in diversity between pairs of records were not expressed in terms of species richness but using the Simpson diversity index, which is less sensitive to the species with low cover. This is justified in order to minimize the influence of a possible bias related to the fact that species with very low cover are mainly those overlooked (Vittoz and Guisan 2007).

Two conversions of Braun‐Blanquet's scale were used for subsequent analyses. The Braun‐Blanquet's scale was converted into the median of the cover class (Table 2), in order to test the changes in the species cover between the different periods. By contrast, for all other analyses (Simpson diversity, Bray–Curtis dissimilarity, PCA, mean ecological values), numerical codes (Gillet 2000) were used because they preserve the importance of the less abundant species, a crucial point in such analyses, by reducing the weight given to dominant ones (high cover).

A possible homogenization in plant composition between historical and recent records in a same vegetation type was tested with the Bray–Curtis dissimilarity. This index computes the beta‐diversity between a given record and all the others during the same time period, considering their respective species composition and cover. Means of dissimilarity indices were computed for each record separately for historical and recent surveys. Pairwise Wilcoxon–Mann–Whitney tests were used to compare temporal differences between medians of Simpson diversity indices and mean Bray–Curtis dissimilarities. The Wilcoxon test was applied firstly in the bilateral mode, and, if it gave a significant result, the unilateral mode was applied as well. The *P*‐values reported in the text refer to the unilateral mode.

The difference between recent and historical species frequencies was calculated and tested with a restricted permutation test following Kapfer et al. (2011) within each plant community. Treating historical and recent inventories separately, the occurrences of each plant species among plots were shuffled randomly 999 times and new frequencies were calculated for each repetition. Significance levels were assessed by counting the number of times the changes in frequency between random historical and recent data was larger or equal to the observed changes in frequency between observed historical and recent data. For the species present simultaneously in at least 25% of the historical and recent inventories, a mean cover was calculated considering only the plots where the species was observed. Changes in mean cover were tested with the same restricted permutation test used for species frequency but using the mean cover values instead (Kapfer et al. 2012).

The floristic shifts between historical and recent records were visualized using two principal component analyses (PCA, R *vegan* library): one based on species composition and cover, and the other based on presence–absence data. The cover values were previously submitted to Hellinger transformation, which is recommended when performing PCA with species cover data (Borcard et al. 2011). In order to test the significance of the temporal shifts in species composition and cover along the first three axes of PCA, a multivariate analysis of variance (MANOVA) was applied on the differences of axis scores against the intercept for each vegetation type individually (Vittoz et al. 2009).

Landolt ecological indicator values (Landolt et al. 2010) were used to investigate which of the environmental factors were related to the changes. These values, which are species specific, vary between 1 and 5 and express increasing species requirements in terms of air temperature (T), light (L), soil humidity (F), soil pH (R), and nutrient content (N). Mean indicator values per plot were calculated with the cover as a weight. Temporal changes of mean indicator values were checked using pairwise Wilcoxon–Mann–Whitney tests. All data processing and analyses were performed with R software, version 3.1.1 (R Core Team, 2014).

## Results

### Distribution among vegetation types

Sixty‐three pairs of reliable records have been retained (Table 1): 10 in the calcareous grasslands, 13 in the windy ridges, 12 in the siliceous subalpine grasslands, 11 in the siliceous alpine grasslands, 8 in the typical snowbeds, and 9 in the wet snowbeds. A clustering analysis (using the Hellinger distance and the Ward aggregation algorithm) of cover‐weighted historical and recent inventories together showed that all old and recent records were placed by pairs in the same group corresponding to their respective plant community, except for one snowbed plot (R3935), which shifted from the wet to the typical snowbeds. For subsequent analyses, this record was retained at its original group.

### Diversity changes

**Figure 3:**
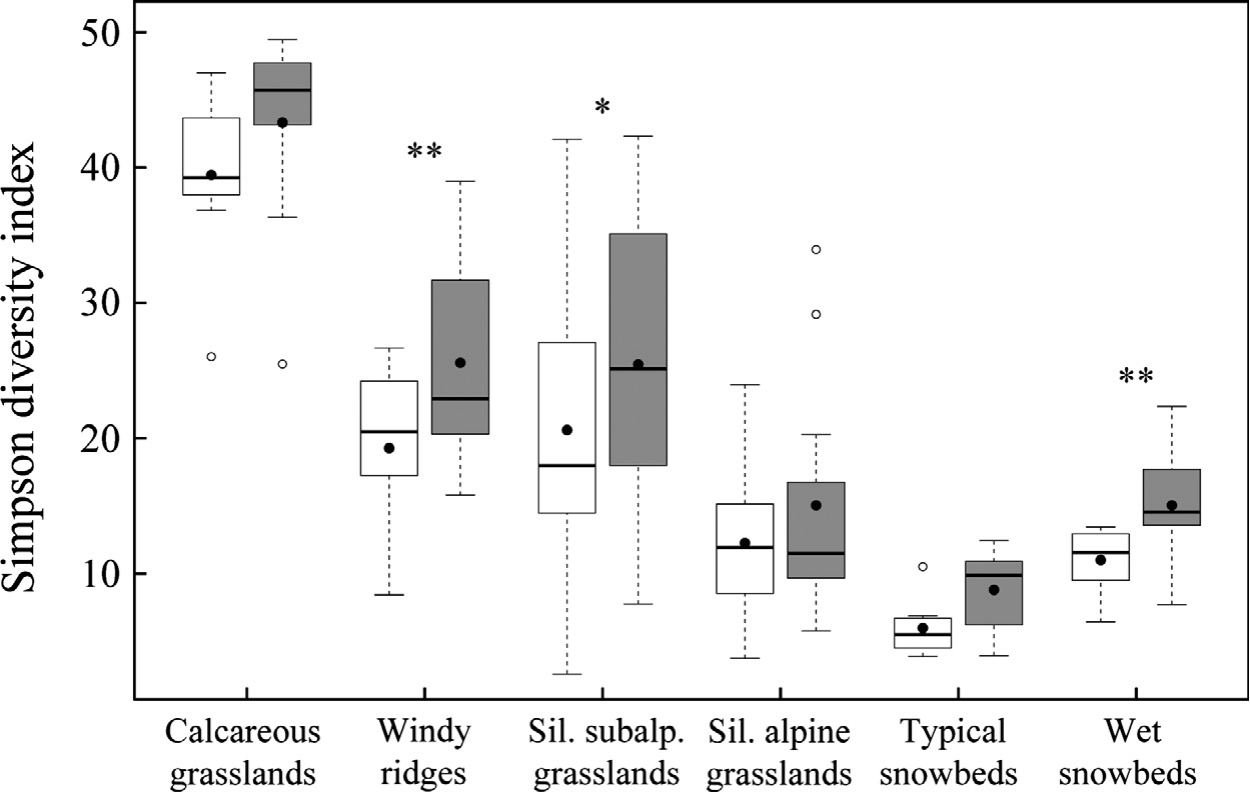
Simpson diversity index for historical (white boxes) and recent (gray boxes) inventories in six plant communities. “Sil.”: siliceous; “subalp.”: subalpine. Black dots represent the mean values, the black line is the median, and boxes are limited by 1st and 3rd quartiles. Stars above the boxes indicate a significant change between historical and recent inventories, according to a pairwise Wilcoxon–Mann–Whitney test: *P < 0.05; **P < 0.01.

Between the historical and the recent surveys, 47 of 63 plots show an increase in alpha‐diversity and 16 show a decrease. The magnitude of the increase varies between vegetation types (Fig. 3). The windy ridges show the highest increase in the mean Simpson diversity index (+6.3 ± 6.0, difference between medians being significant with a *P*‐value = 0.004), followed by the siliceous subalpine grasslands (+4.8 ± 6.7, *P*‐value = 0.017) and the wet snowbeds (+4.1 ± 3.5, *P*‐value = 0.004). The increase in alpha‐diversity in the other plant communities is not significant.

**Figure 4:**
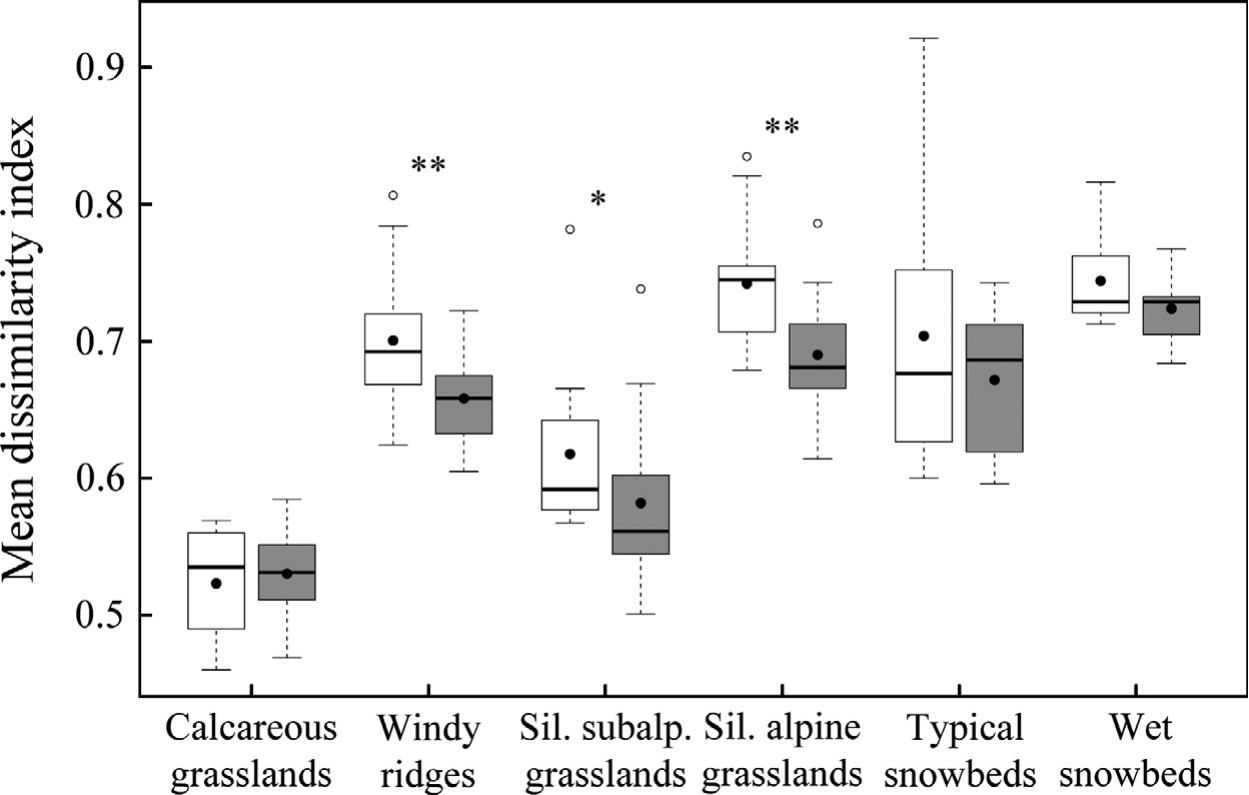
Averages of Bray–Curtis dissimilarity indices among historical (white boxes) and recent (gray boxes) inventories in six plant communities. Same symbols as in Figure 3.

Beta‐diversity shows an opposite trend with a slight decrease in the mean Bray–Curtis dissimilarity index between historical and recent records in each plant community, except for the calcareous grasslands (Fig. 4), whose inventories always show the same low dissimilarity level. The highest homogenization is observed in the siliceous alpine grasslands, where the mean dissimilarity index decreased by 0.05 ± 0.03 (*P*‐value = 0.002), followed by the windy ridges (−0.04 ± 0.04, *P*‐value = 0.002) and the siliceous subalpine grasslands (−0.04 ± 0.04, *P*‐value = 0.010). The two snowbeds also show a dissimilarity decrease, but not significantly.

### Shifts of plant communities

**Figure 5:**
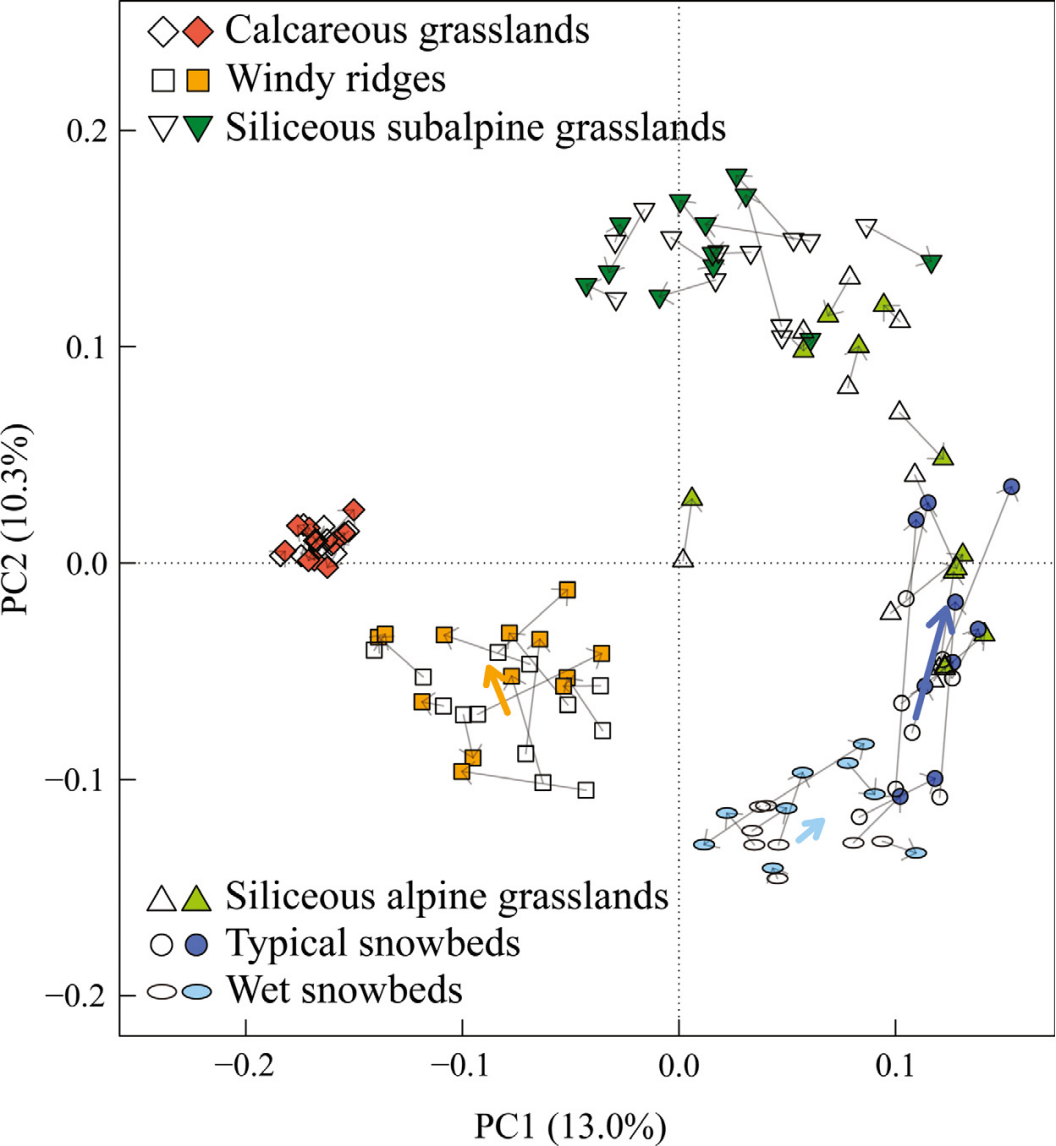
Principal component analysis based on species composition and cover. The first axis represents 13.0% of the variance and the second 10.3%. Couples of historical (empty symbols) and recent (full symbols) records are connected with thin arrows. Thick arrows represent a significant shift of the plant community centroids.

**Figure 6:**
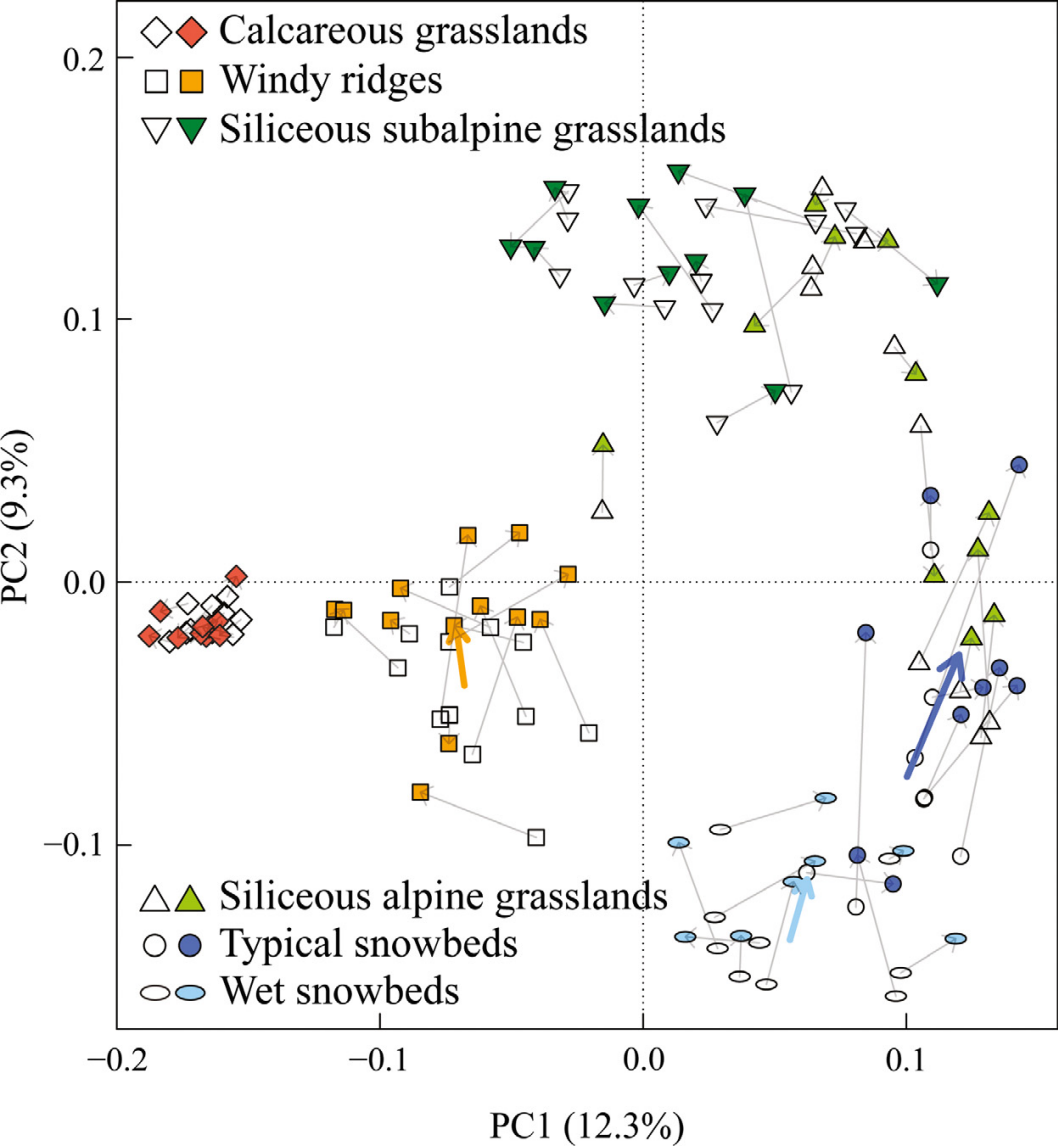
Principal component analysis based on species composition (presence–absence). The first axis represents 12.3% of the variance and the second 9.3%. Same symbols as in Figure 5.

The six plant communities display different directions and amplitudes in their temporal shifts in the cover‐weighted PCA (Fig. 5). The first two axes of PCA explain 23.3% of the total variance (PC1: 13.0%; PC2: 10.3%). The most evident shifts are those of snowbeds: the typical ones show a significant (*P*‐value = 0.012) unidirectional trend toward the siliceous alpine grasslands*,* while the recent species composition of the wet snowbeds is significantly closer (*P*‐value = 0.006) to the typical snowbeds than the historical composition. The windy ridges plots shift in two main directions (*P*‐value = 0.047), either toward calcareous grasslands or the siliceous ones. The three grassland communities have no significant shift in species composition. In particular, the calcareous grasslands display a high stability in terms of species composition. Similar trends, in direction and magnitude, are displayed when presence–absence data are considered (Fig. 6). However, four couples of records originally attributed to the siliceous alpine grasslands are here assimilated to the typical snowbed group, sharing with it the same unidirectional trend toward siliceous grasslands. These records have a species composition similar to those of typical snowbeds, but, because of the dominance of some grassland species, they are assimilated to the alpine grassland group when cover is taken into account. Hence, they can be considered as transition between snowbeds and siliceous alpine grasslands.

### Changes in species frequency and cover

In all the vegetation types but the calcareous grasslands, the number of species, whose frequency increased since the historical survey, exceeds species whose frequency decreased (data available from the Dryad Digital Repository: http://dx.doi.org/10.5061/dryad.q82j0), and only increasing frequencies are significant. Regarding changes in species cover, most of the species in the calcareous grasslands, the siliceous subalpine, and alpine grasslands show a decrease in the mean cover, whereas most of the species in the windy ridges, the typical, and wet snowbeds increase in cover. But very few cover changes are significant.

In the calcareous grasslands, five species with their optimum mostly at the subalpine belt increase significantly: *Festuca ovina aggr*., *Globularia cordifolia*,* Cirsium acaule*,* Plantago atrata s.str*., and *Polygala alpestris*. Interestingly, *Globularia cordifolia*, a typical species of upper montane–lower subalpine belt according to the temperature indicator value (Landolt et al. 2010), was absent in the historical survey, but is present in 50% of the recent plots. *Carex sempervirens* shows a strong decrease in mean cover (−15%, *P*‐value = 0.001). In windy ridges, species from both calcareous (*Anthyllis vulneraria* subsp. *alpestris* and *Selaginella selaginoides*) and siliceous grasslands (*Hieracium angustifolium*), or from the ridge community itself (*Agrostis alpina*) and generalist species (*Campanula scheuchzeri*), display a significant frequency increase.

The occurrence of three subalpine species (*Solidago virgaurea* ssp. *minuta*,* Trifolium pratense* ssp. *nivale*, and *Arnica montana*) is significantly higher in recent siliceous subalpine grassland surveys than in the historical ones. *Nardus stricta* markedly decreases in mean cover (−11.5%, *P*‐value = 0.029). In the siliceous alpine grasslands, four species typical of this community (*Euphrasia minima*,* Agrostis rupestris*,* Homogyne alpina*, and *Hieracium alpinum*) are distributed more widely among recent surveys than in the historical ones.

The species, whose frequency and cover greatly increased in typical snowbeds, are mostly from siliceous alpine grasslands as well: *Leontodon helveticus* increases by 62.5% in frequency (*P*‐value = 0.019) and 3.3% in cover (not significant), while *Helictotrichon versicolor* was absent in the historical survey, but is present in half of the recent plots (marginally significant, *P*‐value = 0.057). Between the other species increasing both in frequency and cover (defined as “winners”, Appendix S3c), most of them are typical of grasslands and are generalists (*Ligusticum mutellina, Nardus stricta)*. In contrast, the species with the most important, but not significant, cover decrease (*Carex foetida*) is typical of snowbeds.

In the wet snowbeds, some species mostly associated to typical snowbeds, such as *Sibbaldia procumbens*, increase in frequency (+55.6%, *P*‐value = 0.019), while *Juncus triglumis*,* Saxifraga androsacea,* and *Gentiana bavarica,* three species growing in wet snowbeds, decrease in terms of mean cover (−26.3%, *P*‐value = 0.008; −18.4%, *P*‐value = 0.026; −15%, *P*‐value = 0.047, respectively).

### Ecological indicator values

**Figure 7:**
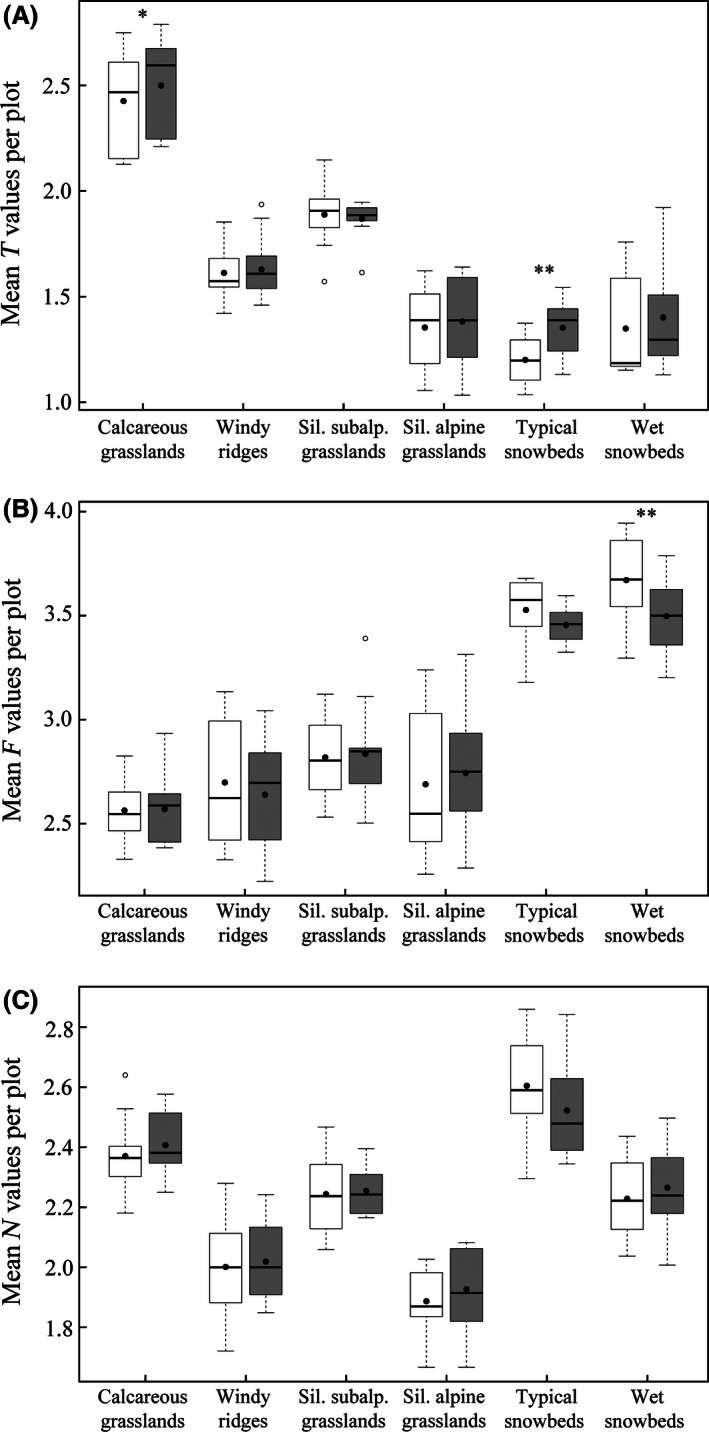
Cover‐weighted means of indicator values (Landolt et al. 2010) for temperature (A), soil humidity (B), and soil nutrient content (C) in historical (white boxes) and recent (gray boxes) inventories. Same symbols as in Figure 3.

The six vegetation types display mean temperature indicator values (Landolt et al. 2010) that reflect their distribution in elevation, with highest values for the calcareous grasslands (Fig. 7A). The calcareous grasslands and the typical snowbeds are the only plant communities showing a significant increase in their mean temperature values between inventories (*P*‐value = 0.010 and *P*‐value = 0.004, respectively). Similarly, the value for soil humidity (F) reflects the moisture conditions of the plant communities, with the four types of grasslands having lower values than the two snowbed communities (Fig. 7B). Species present in the recent records of the typical and wet snowbeds have, on average, lower values than the composition of historical surveys, indicating their preference for drier conditions. However, only the decrease in the latter one is significant (*P*‐value = 0.004). None of the studied plant communities show significant variations between historical and recent surveys in terms of soil nutrient requirements (Fig. 7C), light, and soil pH (Appendices S4 and S5), according to the corresponding mean ecological indicator values.

## Discussion

The results of this study clearly indicate that vegetation changed over a 25‐ to 50‐year time span at the subalpine–alpine level in the Swiss Alps. The six plant communities display similar alpha‐ and beta‐diversity changes, but also various reactions to past environmental changes in terms of species composition.

### Alpha‐ and beta‐diversity

The increase in species richness, expressed as Simpson diversity index at the plot scale, is observed in each plant community. There are three possible explanations: (1) new species arrived since the historical time; (2) the recent inventories were more exhaustive than the historical ones, or (3) the new species are the result of inaccurate location of the plots. The last option can be excluded because it cannot result in a systematic increase for all the vegetation types. The second option could be meaningful only for the least frequent species (i.e., occurring in one or two new plots), but not for those with a considerable increase (for example, *Globularia cordifolia* in the calcareous grasslands). Moreover, many of these species are easily visible in terms of size and/or difficult to confuse with other species. Therefore, the colonization of plots by new species is at least partly responsible for the observed increase in alpha‐diversity. Many previous studies observed the same trend over the last three decades on alpine plant communities (Kudernatsch et al. 2005; Britton et al. 2009; Vittoz et al. 2009; Sandvik and Odland 2014), or even just over 6 years in snowbeds (Carbognani et al. 2014; Pickering et al. 2014). Olsen and Klanderud (2014) observed that species‐poor communities were more susceptible to species invasion than highly diverse species communities. Our results do not confirm such a trend, as the highest species increase was observed on the windy ridges community, which are more diverse than typical snowbeds.

The increase in species richness is related to an increase in the floristic similarity inside the plant community, except in the calcareous grasslands. Similar homogenization was first highlighted on seven European Alpine summits by Jurasinski and Kreyling (2007), and on a variety of alpine plant communities since then (Britton et al. 2009; Ross et al. 2012; Carbognani et al. 2014). According to their observations, the biotic homogenization results from two processes: the invasion of widespread and generalist species, and a decline of rare and specialized species. Generalist species may be able to spread in new areas previously unsuitable, thanks to less constraining conditions for their establishment and survival, such as longer growing seasons through climate warming, or increased nutrient availability (Britton et al. 2009). Indeed, such a pattern is apparent in this study, where snowbed specialists decrease in cover, while grassland generalist species increase in frequency and cover (see Appendix S3). An increasing alpha‐diversity coupled with a homogenization can be explained by the arrival of previously missing species in the community, completing the typical species ensemble for a given vegetation type (e.g., *Agrostis alpina* in the windy ridges, *Arnica montana* in the siliceous subalpine grasslands).

### Snowbeds

The main changes in plant composition are observed in the typical snowbeds, which show a marked shift of species composition and cover toward the siliceous alpine grasslands, and in the wet snowbeds, whose composition tends toward the typical snowbeds (Figs 5 and 6). Therefore, the snowbeds are now more similar to the siliceous alpine grasslands than they were in the 1970s. This is confirmed by the observed colonization by species from siliceous alpine grasslands (*Helictotrichon versicolor*) in the typical snowbeds or their increase in both frequency (*Leontodon helveticus)* and cover (*Nardus stricta*). This expansion of grassland species is reflected in the increase in the temperature indicator value and in the decrease in the humidity one (Fig. 7A,B). These conclusions are consistent with results from previous long‐term monitoring across alpine areas of the Scandes (Virtanen et al. 2003; Kapfer et al. 2012; Sandvik and Odland 2014), Scotland (Britton et al. 2009), Caucasus (Elumeeva et al. 2013), Japan (Kudo et al. 2011), and Greenland (Daniëls et al. 2011).

Similar changes have been observed even on shorter timescales, as in 6‐year surveys from Italy (Carbognani et al. 2014) and Australia (Pickering et al. 2014). All these studies agree that the arrival and expansion of grassland species in the snowbed communities is likely a consequence of longer growing seasons induced by earlier snowmelt dates. The melt‐out date, which is an important driver of arctic and alpine plant growth (Jonas et al. 2008), shifted earlier by 1–4 days per decade between 1998 and 2015 at 2110–2630 m.a.s.l. next to our three study sites (Appendix S6a). This shift, although not significant and covering a short time period, is corroborated by satellite observations in the high‐latitude and high‐elevation areas of the Northern Hemisphere (Dye 2002). This is probably the consequence of two associated factors: firstly, the increase in mean annual temperature, which has been calculated as 1.82 K between 1961 and 2008 in Switzerland (Serquet et al. 2013), which is equivalent to the double of the mean change for the Northern Hemisphere (Rebetez and Reinhard 2008), and secondly, the decrease in the snowfall/precipitation ratio estimated to be around 0.25% per year at the beginning and the end of the snow season from 1961 to 2008 (Serquet et al. 2013). The spring decreasing trend of snowfall/precipitation day ratio has been observed even at 2500 m a.s.l. by Marty and Meister (2012) but is generally more pronounced at lower elevations (Scherrer et al. 2004; Serquet et al. 2013). In the three present study sites, despite a high interannual variability, the annual sum of fresh snow thickness decreased by 0.49–0.96% per year between 1964 and 2011 (Fig. 2). The autumn and spring months seem to be crucial for snow duration, because at that period of the year, air temperatures are closer to the melting point than during the winter (Serquet et al. 2011), and a slight increase is sufficient to reduce the snowfall part of precipitations. The lower snow amount and earlier melting dates observed in the study sites were accompanied by lagged snow falls in autumn (Appendix S6b). The resulting longer growing season (+5 to 14 days per decade between 1998 and 2015, not significant, Appendix S6c) allows the invasion of generally more competitive species, such as graminoids (Dullinger et al. 2007). These species now have enough time to accomplish their life cycle in a snowbed. The establishment of species from adjacent communities could have been enhanced by (1) the proximity of grasslands to snowbeds (mostly <20 m from the study sites), (2) the snowbed potential of trapping seeds (Larsson and Molau 2001), and (3) the high dispersal capacity of certain grassland species. Indeed, the increase in frequency of *Leontodon helveticus* could be associated to its pappus appendage, which was shown to give an advantage to plant species in colonizing new Alpine summits (Matteodo et al. 2013).

Moreover, snow is an efficient scavenger of atmospheric pollutants, which are leached through the snowpack, mainly at the beginning of the melt period (Johannessen and Henriksen 1978). The consequent high load of nitrogen into the snowbed soils can damage certain species (as the moss *Kiaeria starkei*; Woolgrove and Woodin (1996)) and favor the establishment of acquisitive (nutrient‐rich) plants. For example, graminoid cover has been shown to be directly related to nitrogen deposition in acidic grasslands (Dupré et al. 2010). However, an increase in the mean nutrient indicator value (Landolt et al. 2010) that could support this hypothesis has not been observed in the study sites (Fig. 7C). But, we cannot exclude that higher temperatures, combined with relatively high nutrient level in the soil, allow more thermophilous species (grassland species) to establish in the snowbeds, independently from the length of the growing season.

The snowbed species are able to respond positively to experimental warming (Arft et al. 1999; Sandvik and Totland 2000) and can theoretically profit for earlier snow‐free habitats. But they are restricted to snowbed habitats because of lower competition from co‐occurring plants (Heegaard and Vandvik 2004). The arrival of taller species from the surrounding grasslands might increase the competition and induce a decrease in typical snowbed species. Hulber et al. (2011) suggested that the presence of neighbors in snowbed systems leads to competitive effects rather than facilitative ones, which can be expected in such harsh environmental conditions (Choler et al. 2001). Moreover, the role of competition might increase with warming, as experimentally observed by Olsen and Klanderud (2014). In the study sites, no significant decrease is observed, but the strong decrease in cover of *Carex foetida* could be a first sign of such an evolution.

Similar to the typical snowbeds, but over a shorter time period (median of historical records years = 1988, Table 1), the wet snowbeds show increasingly dry conditions. Reductions in snow precipitation, combined with higher temperatures, likely shorten the amount and duration of water supply (Beniston et al. 2003) to these communities, mostly located under melting firn. The cover decrease in typical alliance species and the diffusion of snowbed species, in parallel with the reduction in the mean humidity indicator value (Fig. 7B), indicate that these sites are rapidly shifting toward typical snowbed communities. The same drying trend was observed with the expansion of some graminoids and shrub species in Norwegian wet snowbeds (Sandvik and Odland 2014), on soligenous and ombrogenous mires (Virtanen et al. 2003; Ross et al. 2012), and springs (Britton et al. 2009). These last vegetation types do not belong to snowbeds, but they are subject to the same water‐logged conditions, which limit the growth of taller plants. Diverse alpine plant communities, directly related to high water supply, seem to respond similarly to climate changes.

### Grasslands

In contrast to plant communities related to long snow cover, calcareous and siliceous grasslands demonstrate a high stability of species composition and cover, whatever the bedrock type (Figs 5 and 6). Similar results were obtained by warming experiments on subalpine meadows in the Rocky Mountains (Price and Waser 2000), on calcareous grasslands in northern England after a 13‐years exposure to climate changes (Grime et al. 2008), and observed too by long‐term surveys in the Alps (Vittoz et al. (2009), Windmaißer and Reisch (2013). These authors identified many possible explanatory factors. Firstly, the high plant density and belowground phytomass of subalpine grasslands, compared to the sparse vegetation of alpine and nival summits or to the low species abundance in snowbeds, lead to high competition levels for light and soil resources, which restricts the establishment of new species (Choler et al. 2001). Secondly, the extreme longevity of some grass species (*C. curvula* can reach a maximum of 5000 years; de Witte et al. 2012), the persistence of their shoot and root systems, and their clonal growth, that allows the continuous recolonization of vegetation gaps, result in a high resilience to interannual variations (Hillier et al. 1990) with a consequent long‐term persistence. For example, *Laserpitium siler*, which was a dominant species in half of the plots in calcareous grasslands, is highly competitive in terms of light and water resources and occupies a wide elevation range, thus likely preventing colonization by new species. Thirdly, the steep slopes where the calcareous grasslands are established could also explain their stability. According to Theurillat and Guisan (2001), slopes steeper than 40° (which is often the case in this study) may act as barriers to upward dispersal of species.

Nevertheless, this general stability is also accompanied by new species or increase in frequency. Some of these species (*Globularia cordifolia*,* Cirsium acaule*), although frequently associated to calcareous grasslands, have their optimum at lower elevations. Conversely, the only significantly declining species, *Carex sempervirens,* has its optimum at the lower alpine rather than the subalpine belt. These changes in composition are reflected by a significant increase in the mean indicator value for temperature observed across the calcareous grasslands (Fig. 7A). In conclusion, although displaying a high stability, these grasslands seem to experience the arrival of species from lower elevations, as repeatedly observed on alpine and nival summits (see Stöckli et al. 2011 for a review). Interestingly, in long‐term studies focused on lower elevation grasslands (Britton et al. 2009; Vittoz et al. 2009; Ross et al. 2012; Elumeeva et al. 2013; Windmaißer and Reisch 2013), most of the species decreasing in frequency and/or cover have an alpine‐to‐arctic distribution, while those increasing have broader or lower elevation ranges.

Siliceous subalpine and alpine grasslands show a different trend with supplementary species either having very widespread distribution (*Euphrasia minima*,* Homogyne alpina*) or arriving from the same species pool (*Arnica montana, Hieracium alpinum*). This process, known as range filling, was already observed in the Italian Alps by Cannone and Pignatti (2014) and seems to be predominant compared to the upward shift. Indeed, neither did montane species colonize the siliceous subalpine grasslands, nor did subalpine species move upward and colonize the siliceous alpine grasslands. The abovementioned stabilizing factors appear to be important in these siliceous grasslands.

According to Dullinger et al. (2012), the elevational shift of plant species observed on alpine summits may display faster cool edge expansion than warm edge retreat because of the potentially long persistence of declining populations under unsuitable conditions. The stability of the subalpine and alpine grasslands, while snowbeds are changing, seems to confirm this prediction and indicates that, during the last few decades, subalpine and lower alpine species expanded upwards from their elevational range rather than shifting it.

### Windy ridges

The community on windy ridges shows a significant change in species composition according to the PCA (Figs 5 and 6). Indeed, the centroid shifts toward the calcareous grasslands, although some of the recent inventories are closer to the siliceous grasslands instead. The species increasing in frequency confirm this pattern, with some related to the calcareous grasslands and others to the siliceous ones. The different shifts seem to be related to soil pH, as shown by soil analyses, but a higher number of plots would be necessary for a better understanding of these divergences. Research on comparable habitats (such as alpine heaths on windy ridges) shows diversified reactions to past climatic changes, from very limited changes (Elumeeva et al. 2013), to an increase in dwarf shrubs (Virtanen et al. 2003) or graminoid increase related to a dwarf shrub and forb decrease (Ross et al. 2012). The only common feature is the lichen decrease, attributed either to summer reindeer grazing (Virtanen et al. 2003), or to nitrogen deposition (Armitage et al. 2014), trampling, and climate warming (see Ross et al. 2012 and references therein). Unfortunately, the majority of our historical inventories do not give any indication of lichen covers (Appendix S2). Consequently, this study cannot confirm such a trend.

### Long‐term implications

This study is the first of its kind to assess the way different plant communities in the subalpine and lower alpine belts of the European Alps reacted to climate changes over the last two to four decades. It demonstrates that reactions differ considerably between vegetation types, with the most important changes in those linked to long snow cover. The vulnerability of *Salicion herbaceae* (typical snowbeds) was already suspected by Braun‐Blanquet (1975). Indeed, monitoring eastern Switzerland vegetation of a very late snowmelt patch dominated by the moss *Polytrichum sexangulare* from 1921 to 1947, Braun‐Blanquet (1975) observed an increasing cover of snowbed plant species in response to shorter snow cover and warmer temperatures. Moreover, he hypothesized that snowbeds will be progressively invaded by species from the surrounding siliceous grasslands. Therefore, it is likely that, during the last few decades, some snowbed communities took refuge in *Polytrichum sexangulare* communities, altering their species composition. Simultaneously, snowbed species colonized many summits and slopes, where, as a result of glacier and snow cover reductions, new snowbed areas were available for colonization (Grytnes et al. 2014). Therefore, snowbed species can still find suitable areas in the coldest microhabitats, but with potential detrimental consequences for the communities currently present. This corroborates the theory of Scherrer and Körner (2011), who sustained that alpine terrain offers a variety of thermal microhabitats over very short distances, which will be suitable for the majority of species.

Beniston et al. (2003) predicted that, with a temperature rise of 4°C in 2071–2100 (Christensen et al. 2002), the snow volume in the Alps at 2000 m may reduce by 50% and the melting season advanced by 50–60 days. As this study clearly demonstrates, changes in snow precipitations may have a stronger impact on the subalpine–alpine plant communities than warmer temperatures, at least for communities directly dependent on snow cover as a limit to the growing season. However, very probably, the grasslands will not be able to stand such a temperature increase without important changes as well. But, with the available data, it is not possible to conclude whether changes will still be very slow, like those observed until now, which will induce a large local extinction debt (Dullinger et al. 2012), or whether strong and sudden changes are expected after forest colonization, successive years of drought, development of diseases (Ayres and Lombardero 2000), or the arrival of new herbivores (Pellissier et al. 2014). Future monitoring of alpine grasslands will be particularly important to address these questions.

## Conflict of Interest

None declared.

## Supporting information


**Appendix S1.** Mean summer temperature variations in the three study sites.
**Appendix S2.** Characteristics of the 126 inventories.
**Appendix S3.** Relative change in cover versus relative change in frequency of the most frequent species.
**Appendix S4.** Cover‐weighted means of indicator values for light (L).
**Appendix S5.** Cover‐weighted means of indicator values for soil pH (R).
**Appendix S6.** Variations of the last snow day, first snow day, and growing season length in the three study sites.
**Appendix S7.** Principal component analysis based on species composition and cover of the calcareous plant communities.
**Appendix S8.** Principal component analysis based on species composition and cover of the siliceous plant communities.
