# Supplementary material for: Snowbeds are more affected than other subalpine–alpine plant communities by climate change in the Swiss Alps
Source: Ecol Evol. 2016 Sep 9;6(19):6969–82. doi: 10.1002/ece3.2354 (PMC5513224; doi:10.1002/ece3.2354)
Supplement: Supplementary file 1 — Appendix S1. Mean summer temperature variations in the three study sites. Appendix S2. Characteristics of the 126 inventories. Appendix S3. Relative change in cover versus relative change in frequency of the most frequent species. Appendix S4. Cover‐weighted means of indicator values for light (L). Appendix S5. Cover‐weighted means of indicator values for soil pH (R). Appendix S6. Variations of the last snow day, first snow day, and growing season length in the three study sites. Appendix S7. Principal component analysis based on species composition and cover of the calcareous plant communities. Appendix S8. Principal component analysis based on species composition and cover of the siliceous plant communities. [file ECE3-6-6969-s001.docx]

**Appendix 1.** Mean summer temperatures (from June to September) during the 1950-2013 period at Château-d’Oex (CHD) and Grimsel Hospiz (GRH) meteorological stations and from 1987 to 2013 at Evolène (EVO). All the stations belong to the MeteoSwiss network (Begert *et al.*, 2005)

**
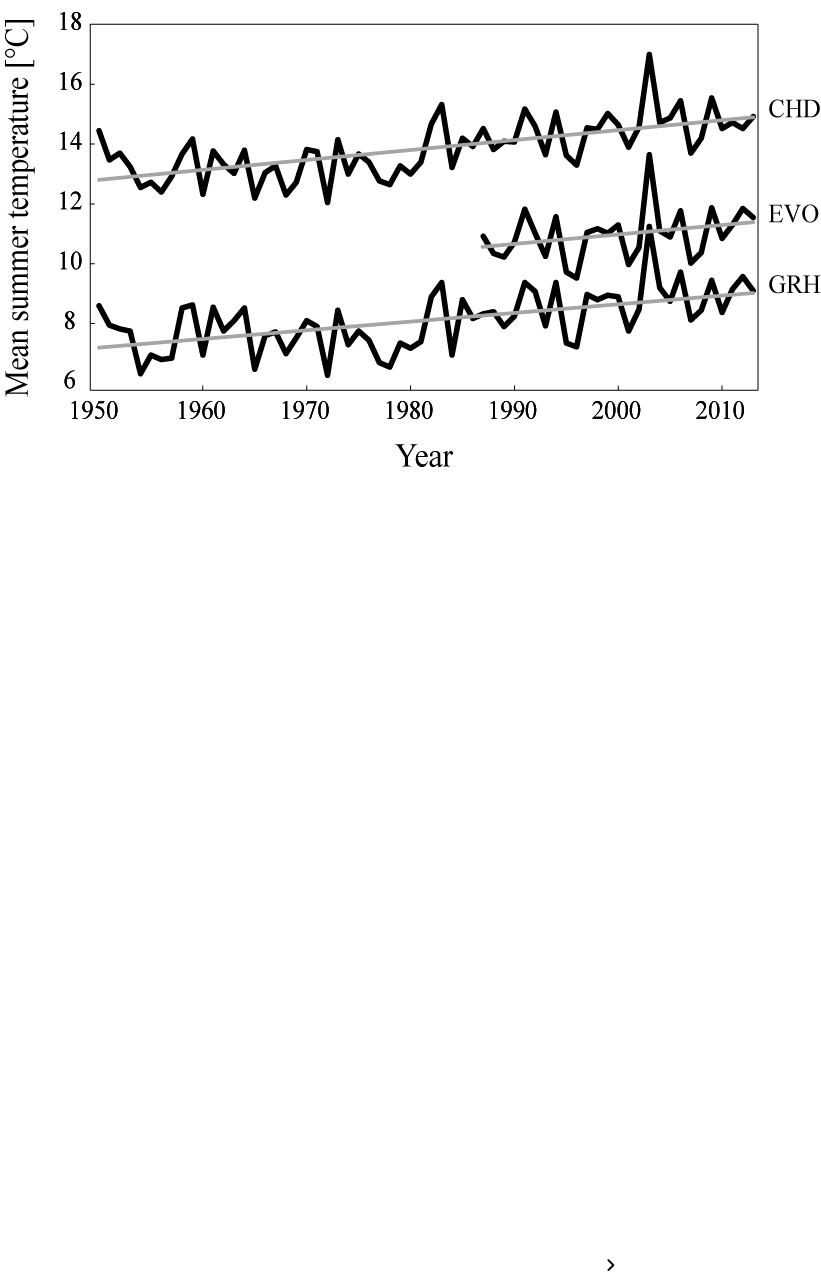
**

**Appendix 2.** List of the 126 inventories (63 historical and 63 recent) included in this study, with the corresponding author's names, time span of survey, spatial coordinates in Km (according to the Swiss reference system for geographical coordinates, CH1903+), elevation, plant community (phytosociological alliance), herb, bryophyte and/or lichen, litter, rock and bare soil covers, and current land use. Covers are given in %, except values with * for which the Braun-Blanquet's scale was used. Authors names are abbreviated as follows: Jean-Louis Richard (JLR), Klaus Ammann (KA), Benoît Bressoud (BB), Olivier Duckert (OD), Magalì Matteodo (MM), Pascal Vittoz (PV), Marie-José Petétot (MJP), Loïc Liberati (LL), and Swanee Messerli (SM).

| **Plot name** | **Author(s)** | **Date** | **CoordX [Km]** | **CoordY [Km]** | **Elevation [m]** | **Plant community** | **Herbs [%]** | **Mosses & lichens [%]** | **Lichens** | **Litter [%]** | **Rocks [%]** | **Bare soil [%]** | **Currently grazed** |
| --- | --- | --- | --- | --- | --- | --- | --- | --- | --- | --- | --- | --- | --- |
| M2844_1 | JLR | 1972 |  |  |  | Seslerion |  |  |  |  |  |  |  |
| M2844_2 | PV & MM | 2013 | 580 | 155 | 1698 | Seslerion | 90 | 0 |  | 1 | 6 | 3 | no |
| M3110_1 | JLR | 1973 |  |  |  | Seslerion |  |  |  |  |  |  |  |
| M3110_2 | PV & MM | 2013 | 578 | 154 | 1926 | Seslerion | 94.9 | 0.1 |  | 1 | 2 | 2 | yes |
| M3126_1 | JLR | 1973 |  |  |  | Seslerion |  |  |  |  |  |  |  |
| M3126_2 | PV & MM | 2013 | 580 | 155 | 1716 | Seslerion | 89 | 1 |  | 2 | 8 | 0 | no |
| M3127_1 | JLR | 1973 |  |  |  | Seslerion |  |  |  |  |  |  |  |
| M3127_2 | PV & MM | 2013 | 580 | 155 | 1738 | Seslerion | 92 | 0.5 |  | 3 | 4 | 0.5 | no |
| M3128_1 | JLR | 1973 |  |  |  | Seslerion |  |  |  |  |  |  |  |
| M3128_2 | MM | 2013 | 580 | 156 | 1843 | Seslerion | 85 | 1 |  | 3 | 8 | 3 | no |
| M3138_1 | JLR | 1973 |  |  |  | Seslerion |  |  |  |  |  |  |  |
| M3138_2 | PV & MJP | 2013 | 579 | 153 | 1715 | Seslerion | 90.9 | 0.1 |  | 3 | 5 | 1 | no |
| M3139_1 | JLR | 1973 |  |  |  | Seslerion | 100 |  |  |  |  |  |  |
| M3139_2 | PV & MJP | 2013 | 579 | 153 | 1771 | Seslerion | 96.5 | 0 |  | 2 | 1 | 0.5 | no |
| M3140_1 | JLR | 1973 |  |  |  | Seslerion | 80 |  |  |  |  |  |  |
| M3140_2 | PV & MM | 2013 | 579 | 153 | 1997 | Seslerion | 75 | 0 |  | 7 | 13 | 5 | no |
| M3141_1 | JLR | 1973 |  |  |  | Seslerion | 100 |  |  |  |  |  |  |
| M3141_2 | PV & MM | 2013 | 579 | 152 | 2099 | Seslerion | 95.9 | 0 |  | 4 | 0 | 0.1 | no |
| M3150_1 | JLR | 1973 |  |  |  | Seslerion | 100 |  |  |  |  |  |  |
| M3150_2 | PV & MJP | 2013 | 579 | 154 | 1951 | Seslerion | 93 | 0.1 |  | 4.5 | 2 | 0.4 | no |
| M3592_1 | JLR | 1975 |  |  |  | Elynion | 90 |  |  |  |  |  |  |
| M3592_2 | MM | 2013 | 577 | 152 | 2232 | Elynion | 70 | 7.5 |  | 0.5 | 20 | 2 | no |
| M4121_1 | JLR | 1979 |  |  |  | Elynion | 70 |  |  |  |  |  |  |
| M4121_2 | PV & MM | 2013 | 578 | 154 | 2180 | Elynion | 65 | 7 |  | 2 | 33 | 3 | no |
| R239_1 | BB | 1990 |  |  |  | Elynion | 85 |  |  |  |  |  |  |
| R239_2 | MM | 2014 | 604 | 115 | 2550 | Elynion | 75 | 1.5 |  | 0.5 | 22 | 1 | no |
| R331_1 | OD | 1990 |  |  |  | Elynion | 30 | 10 |  |  |  |  |  |
| R331_2 | MM & PV | 2014 | 605 | 117 | 2328 | Elynion | 77 | 10 |  | 6 | 2 | 5 | no |
| R3901_1 | JLR | 1977 |  |  |  | Elynion | 90 |  |  |  |  |  |  |
| R3901_2 | MM | 2014 | 603 | 113 | 2697 | Elynion | 80 | 16 |  | 2 | 1.5 | 0.5 | no |
| R4003_1 | JLR | 1978 |  |  |  | Elynion | 100 |  |  |  |  |  |  |
| R4003_2 | PV | 2014 | 607 | 116 | 2595 | Elynion | 88 | 3 |  | 6 | 1 | 2 | yes |
| R4005_1 | JLR | 1978 |  |  |  | Elynion | 30 |  |  |  |  |  |  |
| R4005_2 | MM & PV | 2014 | 607 | 116 | 2694 | Elynion | 81 | 8 |  | 8 | 2 | 1 | no |
| R4224_1 | JLR | 1979 |  |  |  | Elynion | 90 |  |  |  |  |  |  |
| R4224_2 | MM & PV | 2014 | 604 | 110 | 2386 | Elynion | 80 | 6 |  | 8 | 2 | 4 | yes |
| R4225_1 | JLR | 1979 |  |  |  | Elynion | 80 |  |  |  |  |  |  |
| R4225_2 | PV | 2014 | 604 | 109 | 2400 | Elynion | 85 | 1 |  | 7.9 | 0.1 | 6 | yes |
| R4472_1 | JLR | 1981 |  |  |  | Elynion | 90 |  |  |  |  |  |  |
| R4472_2 | MM & SM | 2014 | 603 | 113 | 2694 | Elynion | 86.5 | 7 |  | 1 | 5 | 0.5 | no |
| R4482_1 | JLR | 1981 |  |  |  | Elynion |  |  |  |  |  |  |  |
| R4482_2 | PV | 2014 | 604 | 109 | 2450 | Elynion | 75 | 7 |  | 10 | 2 | 6 | yes |
| R5066_1 | JLR | 1989 |  |  |  | Elynion | 60 | 50 |  |  |  |  |  |
| R5066_2 | MM | 2014 | 605 | 114 | 2422 | Elynion | 70 | 15 |  | 1 | 5 | 0 | yes |
| R5145_1 | JLR | 1990 |  |  |  | Elynion | 80 |  |  |  |  |  |  |
| R5145_2 | MM | 2014 | 606 | 118 | 2430 | Elynion | 80 | 8 |  | 2 | 10 | 0 | NA |
| G14_1 | KA | 1964 |  |  |  | Nardion | 5* | 1* | +* |  |  |  |  |
| G14_2 | PV, MM, LL | 2013 | 663 | 155 | 2320 | Nardion | 92 | 2 |  | 4 | 1 | 1 | no |
| G281_1 | KA | 1967 |  |  |  | Nardion | 5* | 1-2* | 1* |  | 1* |  |  |
| G281_2 | MM & LL | 2013 | 662 | 155 | 2320 | Nardion | 75 | 7 |  | 3 | 15 | 0 | no |
| G288_1 | KA | 1967 |  |  |  | Nardion | 5* | 1* | +* |  |  | 1* |  |
| G288_2 | MM & LL | 2013 | 662 | 155 | 2370 | Nardion | 50 | 15 |  | 34 | 1 | 0 | no |
| G291_1 | KA | 1967 |  |  |  | Nardion | 5* | +* |  |  |  |  |  |
| G291_2 | PV & LL | 2013 | 662 | 155 | 2348 | Nardion | 85 | 3 |  | 7 | 4 | 1 | no |
| G294_1 | KA | 1967 |  |  |  | Nardion | 5* | +* |  |  |  | +* |  |
| G294_2 | PV, MM, LL | 2013 | 663 | 155 | 2320 | Nardion | 92 | 1.5 |  | 3 | 2 | 1.5 | no |
| G295_1 | KA | 1967 |  |  |  | Nardion | 5* | +* | +* |  |  | +* |  |
| G295 bis_2 | PV, MM, LL | 2013 | 663 | 155 | 2329 | Nardion | 95 | 0.5 |  | 3 | 1 | 0.5 | no |
| G343_1 | KA | 1972 |  |  |  | Nardion | 5* | 1* | 1* |  | 1* | 1* |  |
| G343_2 | MM & LL | 2013 | 662 | 155 | 2330 | Nardion | 65 | 15 |  | 3 | 10 | 7 | no |
| G350_1 | KA | 1972 |  |  |  | Nardion | 3* | +* | +* |  |  |  |  |
| G350_2 | MM & LL | 2013 | 662 | 155 | 2326 | Nardion | 60 | 10 |  | 3 | 25 | 2 | no |
| G351_1 | KA | 1972 |  |  |  | Nardion | 3* | 1* | +* |  | 2* | 1* |  |
| G351_2 | MM & LL | 2013 | 662 | 155 | 2318 | Nardion | 65 | 5 |  | 4 | 25 | 1 | no |
| G352_1 | KA | 1972 |  |  |  | Nardion | 5* | +* | +* |  | +* | +* |  |
| G352_2 | MM & LL | 2013 | 662 | 155 | 2320 | Nardion | 80 | 3 |  | 7 | 10 | 0 | no |
| G421_1 | KA | 1973 |  |  |  | Nardion | 5* | 1* | +* |  | 1* | 1* |  |
| G421_2 | MM & LL | 2013 | 662 | 155 | 2312 | Nardion | 60 | 3 |  | 10 | 20 | 7 | no |
| G47_1 | KA | 1964 |  |  |  | Nardion | 5* | 1* | 1* |  |  | 1* |  |
| G47_2 | MM | 2013 | 663 | 155 | 2317 | Nardion | 78 | 2 |  | 3 | 15 | 2 | no |
| G334_1 | KA | 1970 |  |  |  | Caricion curvulae | 5* | 1* | 1* |  | 1* | 1* |  |
| G334_2 | PV, LL | 2013 | 667 | 155 | 2310 | Caricion curvulae | 83 | 4 |  | 6 | 5 | 2 | yes |
| G335_1 | KA | 1970 |  |  |  | Caricion curvulae | 5* | 1* | 1* |  | +* |  |  |
| G335_2 | PV, MM, LL | 2013 | 667 | 155 | 2410 | Caricion curvulae | 86 | 4 |  | 3 | 5 | 2 | yes |
| G337_1 | KA | 1970 |  |  |  | Caricion curvulae | 5* | 1* | 1* |  | 1* |  |  |
| G337_2 | PV & LL | 2013 | 667 | 155 | 2500 | Caricion curvulae | 50 | 36 |  | 2 | 10 | 2 | yes |
| G338_1 | KA | 1970 |  |  |  | Caricion curvulae | 4* | 1-2* | 2* |  | 1* |  |  |
| G338_2 | PV, MM, LL | 2013 | 666 | 155 | 2603 | Caricion curvulae | 22 | 15 |  | 1 | 60 | 2 | no |
| G339_1 | KA | 1970 |  |  |  | Caricion curvulae | 2* | 2* | 1* |  | 3* |  |  |
| G339_2 | PV & LL | 2013 | 666 | 155 | 2650 | Caricion curvulae | 30 | 19 |  | 1 | 15 | 35 | no |
| G340_1 | KA | 1970 |  |  |  | Caricion curvulae |  |  |  |  |  |  |  |
| G340_2 | PV, LL | 2013 | 666 | 155 | 2560 | Caricion curvulae | 30 | 25 |  | 15 | 30 | 0 | no |
| G90_1 | KA | 1965 |  |  |  | Caricion curvulae | 4* | 1* | 2* |  |  |  |  |
| G90 R_2 | MM | 2013 | 665 | 155 | 2441 | Caricion curvulae | 70 | 12 |  | 5 | 5 | 8 | no |
| G92_1 | KA | 1965 |  |  |  | Caricion curvulae | 2* | 4-5* |  |  | 1* |  |  |
| G92_2 | MM & LL | 2013 | 664 | 155 | 2511 | Caricion curvulae | 60 | 20 |  | 0 | 10 | 10 | no |
| R4209_1 | JLR | 1979 |  |  |  | Caricion curvulae | 80 | several lichens |  |  |  |  |  |
| R4209_2 | MM & SM | 2014 | 603 | 113 | 2581 | Caricion curvulae | 75 | 19.5 |  | 1 | 4 | 0.5 | no |
| R4471_1 | JLR | 1981 |  |  |  | Caricion curvulae | 70 |  |  |  |  |  |  |
| R4471_2 | MM & SM | 2014 | 604 | 113 | 2682 | Caricion curvulae | 63 | 15 |  | 1 | 20 | 1 | no |
| R5067_1 | JLR | 1989 |  |  |  | Caricion curvulae | 70 |  |  |  |  |  |  |
| R5067_2 | MM | 2014 | 604 | 115 | 2528 | Caricion curvulae | 74.5 | 20 |  | 2 | 3 | 0.5 | yes |
| G336_1 | KA | 1970 |  |  |  | Salicion herbaceae | +* | 5* | r* |  |  |  |  |
| G336_2 | PV, LL | 2013 | 667 | 155 | 2497 | Salicion herbaceae | 10 | 68 |  | 15 | 3 | 4 | yes |
| G341_1 | KA | 1971 |  |  |  | Salicion herbaceae | 5-4* | 1-2* |  |  |  |  |  |
| G341_2 | PV, MM, LL | 2013 | 666 | 156 | 2460 | Salicion herbaceae | 88 | 6 |  | 2 | 0 | 4 | NA |
| G404_1 | KA | 1973 |  |  |  | Salicion herbaceae | 5* | 1* |  |  |  |  |  |
| G404_2 | MM | 2013 | 663 | 155 | 2313 | Salicion herbaceae | 75 | 15 |  | 5 | 2 | 3 | no |
| G405_1 | KA | 1973 |  |  |  | Salicion herbaceae | 5* | 1-2* |  |  |  |  |  |
| G405_2 | PV, MM, LL | 2013 | 663 | 155 | 2313 | Salicion herbaceae | 85 | 11.9 |  | 3 | 0.1 | 0 | no |
| G417_1 | KA | 1973 |  |  |  | Salicion herbaceae | 4-5* | +* |  |  | 2* |  |  |
| G417_2 | PV, MM, LL | 2013 | 662 | 155 | 2315 | Salicion herbaceae | 73 | 0 |  | 7 | 20 | 0 | no |
| R264_1 | BB | 1979 |  |  |  | Salicion herbaceae | 100 | 10 |  |  |  |  |  |
| R264R_2 | MM | 2014 | 604 | 114 | 2489 | Salicion herbaceae | 70 | 27 |  | 2 | 1 | 1 | yes |
| R267_1 | BB | 1979 |  |  |  | Salicion herbaceae | 90 |  |  |  |  |  |  |
| R267R_2 | MM | 2014 | 604 | 113 | 2685 | Salicion herbaceae | 80 | 5 |  | 15 | 0 | 0 | no |
| R3935_2 | MM | 2014 | 604 | 115 | 2468 | Salicion herbaceae | 50 | 37 |  | 8 | 2 | 3 | yes |
| R4468_1 | JLR | 1981 |  |  |  | Salicion herbaceae | 80 |  |  |  |  |  |  |
| R4468_2 | PV | 2014 | 604 | 115 | 2440 | Salicion herbaceae | 70 | 10 |  | 20 | 0 | 0 | yes |
| R3934_1 | JLR | 1977 |  |  |  | Caricion bicolori-atrofuscae | 50 |  |  |  |  |  |  |
| R3934_2 | MM | 2014 | 604 | 115 | 2468 | Caricion bicolori-atrofuscae | 80 | 15 |  | 0 | 1 | 4 | yes |
| R3935_1 | JLR | 1977 |  |  |  | Caricion bicolori-atrofuscae | 25 | 100 |  |  |  |  |  |
| R3937_1 | JLR | 1977 |  |  |  | Caricion bicolori-atrofuscae | 20 |  |  |  |  |  |  |
| R3937_2 | MM | 2014 | 604 | 113 | 2569 | Caricion bicolori-atrofuscae | 67 | 30 |  | 1 | 2 | 0 | no |
| R4216_1 | JLR | 1979 |  |  |  | Caricion bicolori-atrofuscae | 25 | 30 |  |  |  |  |  |
| R4216_2 | MM & SM | 2014 | 604 | 113 | 2567 | Caricion bicolori-atrofuscae | 35 | 52 |  | 0.5 | 2.5 | 10 | no |
| R4983_1 | JLR | 1988 |  |  |  | Caricion bicolori-atrofuscae | 20 | 80 |  |  |  |  |  |
| R4983_2 | MM | 2014 | 605 | 113 | 2659 | Caricion bicolori-atrofuscae | 24 | 25 |  | 1 | 50 | 0 | no |
| R5061_1 | JLR | 1989 |  |  |  | Caricion bicolori-atrofuscae | 70 | 75 |  |  |  |  |  |
| R5061_2 | MM | 2014 | 605 | 113 | 2650 | Caricion bicolori-atrofuscae | 70 | 24 |  | 2 | 3 | 1 | no |
| R5062_1 | JLR | 1989 |  |  |  | Caricion bicolori-atrofuscae |  |  |  |  |  |  |  |
| R5062_2 | MM | 2014 | 605 | 113 | 2677 | Caricion bicolori-atrofuscae | 25 | 15 |  | 0.2 | 59.8 | 0 | no |
| R5069_1 | JLR | 1989 |  |  |  | Caricion bicolori-atrofuscae | 10 | 60 |  |  |  |  |  |
| R5069_2 | MM | 2014 | 604 | 115 | 2602 | Caricion bicolori-atrofuscae | 25 | 22 |  | 2 | 50 | 1 | yes |
| R5141_1 | JLR | 1990 |  |  |  | Caricion bicolori-atrofuscae | 70 |  |  |  |  |  |  |
| R5141_2 | MM & SM | 2014 | 605 | 114 | 2567 | Caricion bicolori-atrofuscae | 65 | 9.5 |  | 0.5 | 25 | 0 | no |

**Appendix 3**. Relative change in cover vs. relative change in frequency of the species occurring in at least 20% of the historical or recent records (frequent species). The relative cover and frequency of the frequent species are calculated separately for (a) calcareous grasslands and windy ridges (*Seslerion* and *Elynion*), (b) subalpine and alpine siliceous grasslands (*Nardion* and *Caricion* *curvulae*), and (c) typical and wet snowbeds (*Salicion herbaceae* and *Caricion bicolori-atrofuscae*). Dotted lines represent an arbitrary thereshold of ±1% in cover and frequency changes. Only the plant species above and below this threshold are taken into account in order to focus on the main changes.

Plant species have been splitted into generalist and specialist ones based on Delarze and Gonseth (2008). This publication assigns to each Swiss plant species one or more natural habitats, from which the species is typical of. We defined a plant species as a specialist when only one habitat type was associated to it (filled circles), otherwise it is a generalist (hollow circles). Species names are provided only for winning (++) and losing (--) species.


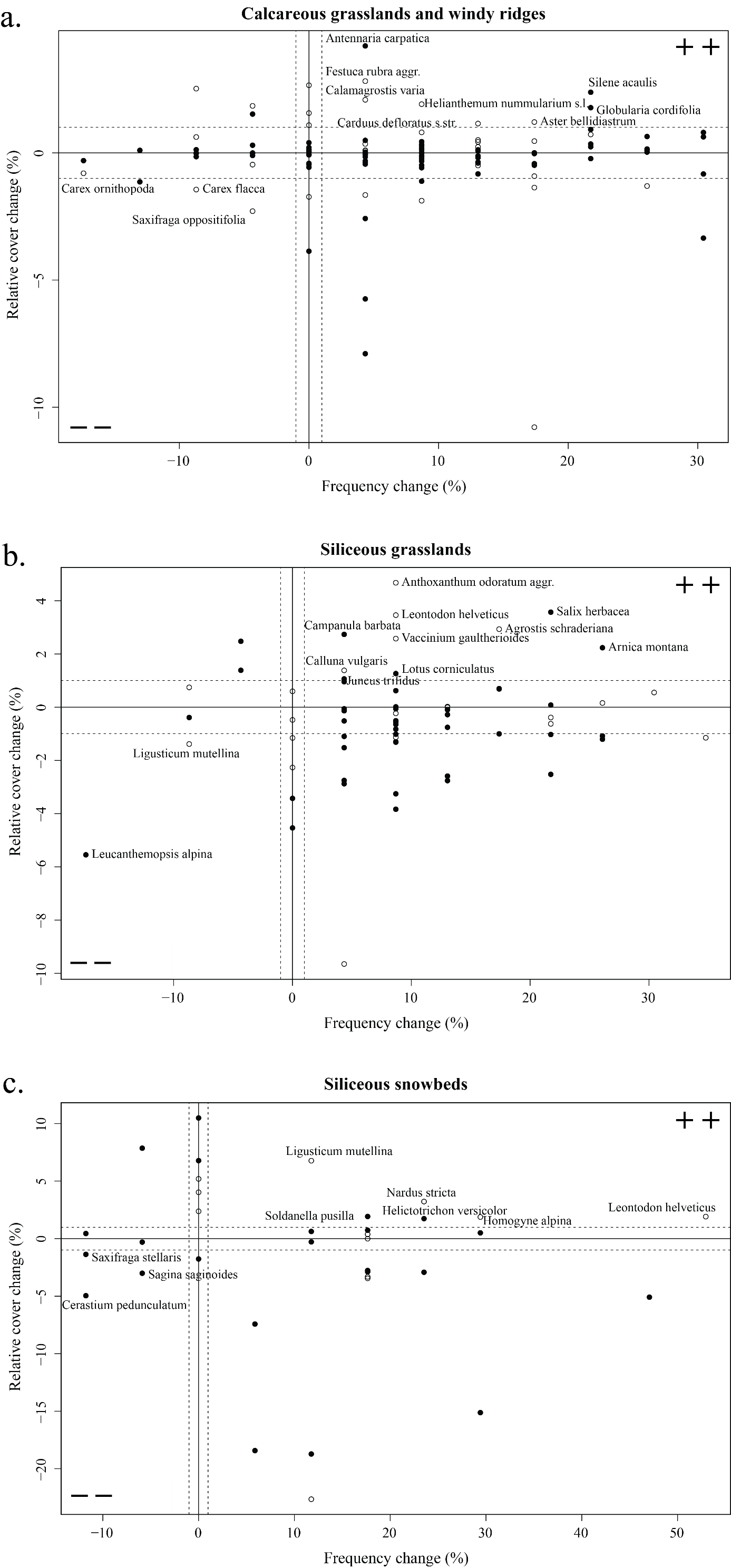


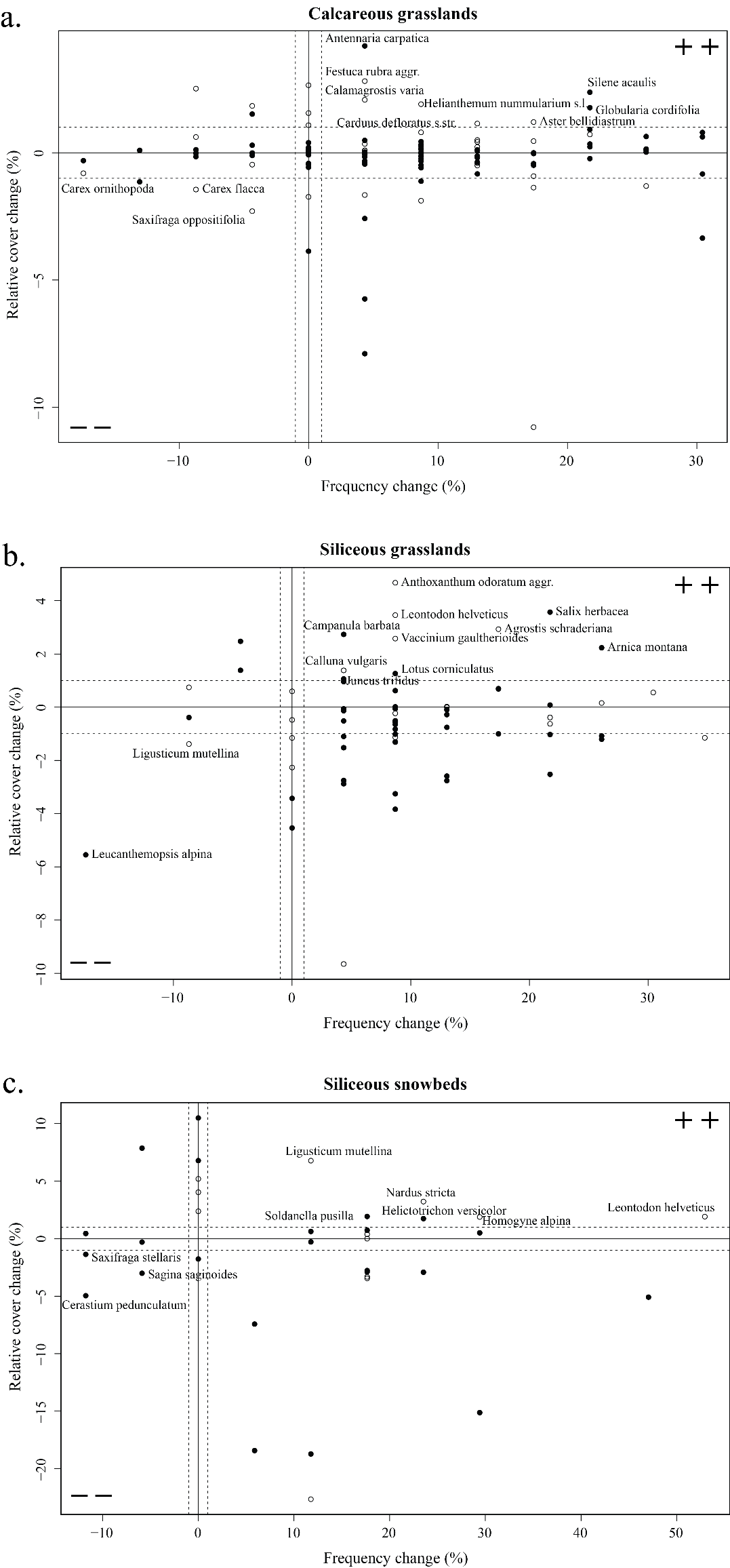


**Appendix 4.**Cover-weighted means of indicator values (Landolt et al., 2010) for light (L) in historical (white boxes) and recent (grey boxes) inventories for each plant community. “Sil.”: Siliceous; “subalp.”: subalpine. Black dots represent the mean values, the black line is the median and boxes are limited by 1^st^ and 3^rd^ quartiles. None of the change is significant with a pairwise Wilcoxon-Mann-Whitney test.


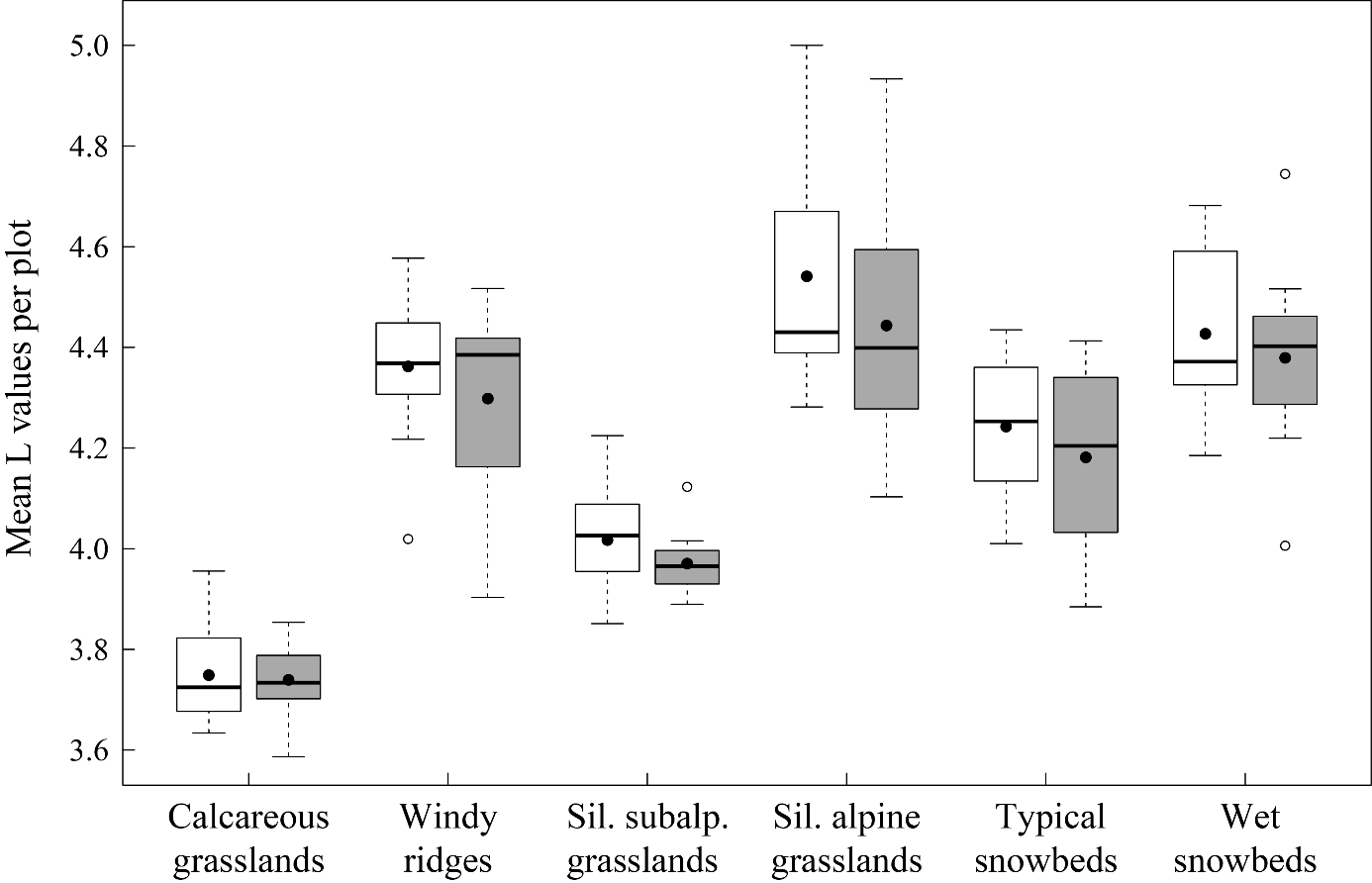


**Appendix 5.** Cover-weighted means of indicator values (Landolt et al., 2010) for soil pH (R) in historical (white boxes) and recent (grey boxes) inventories for each plant community. Symbols are the same as in Appendix 4.


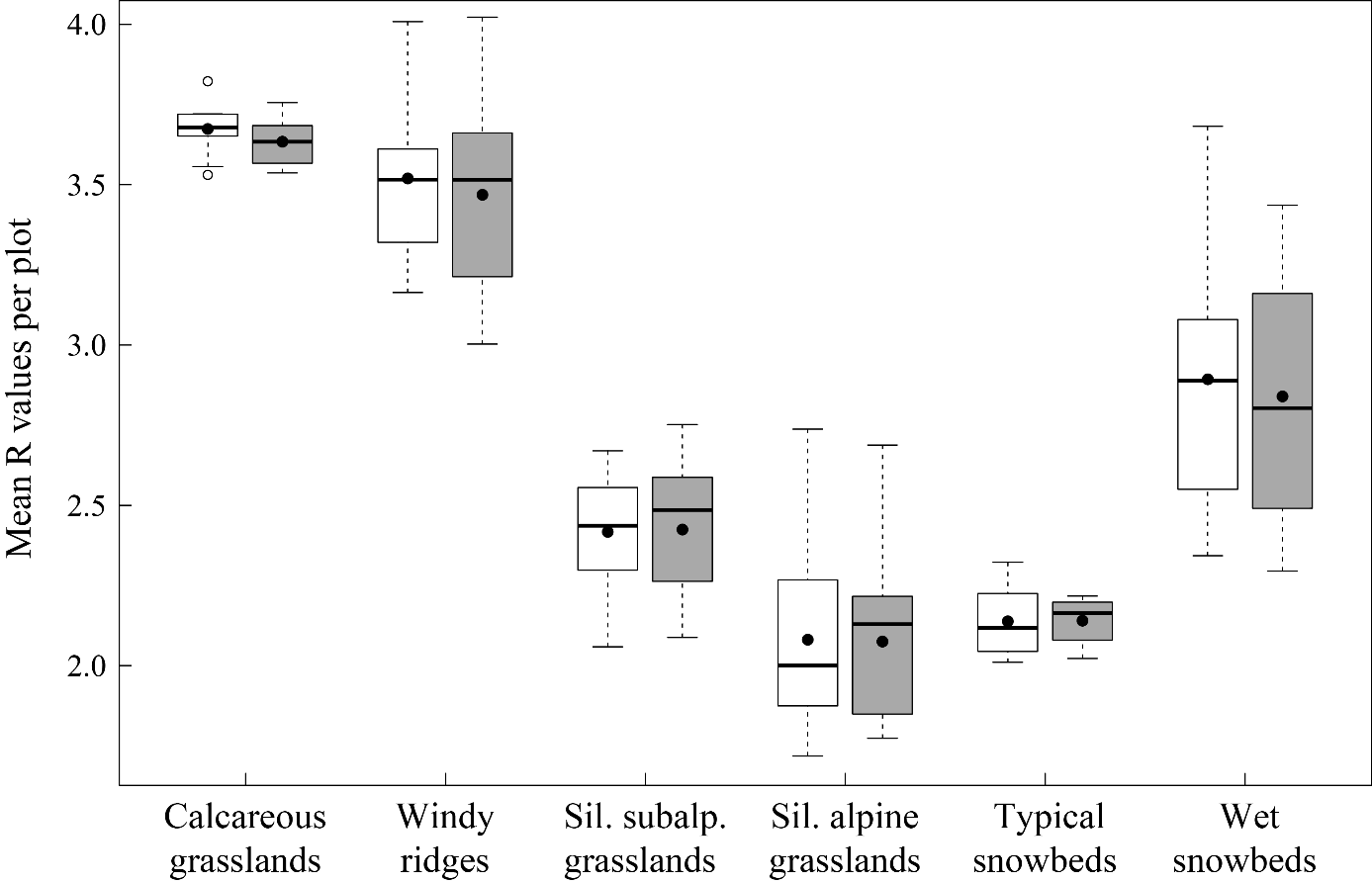


**Appendix 6.** Recent variation of the last snow day (a), first snow day (b) and growing season length (c) on Anniviers meteorological station (ANV, 2630 m a.s.l.; period 1998-2015), on the Oberwald one (OBW, 2430 m a.s.l.; period 2000-2015) and on the Ober Meiel (OBM, 2110 m a.s.l.; period 2001-2015), respectively close to the study sites of Rechy, Grimsel and Morteys. All the stations belong to the WSL Institute for Snow and Avalanche Research SLF (SLF-Messdaten © 2016). Days are calculated from the 1^st^ of January. The “last snow day” is defined as the last day of the winter season with snow cover, the “first snow day” as the first day of the fall season with snow cover and followed by at least one week of continuous snow cover. The growing season length is the difference between the “first snow day” and the “last snow day”. The time series of these three parameters was tested for each station separately with ANOVA, and for all the stations combined with ANCOVA. No significant changes of the “last snow day”, “first snow day” and “growing season length” were detected over these 15-18 years.

**
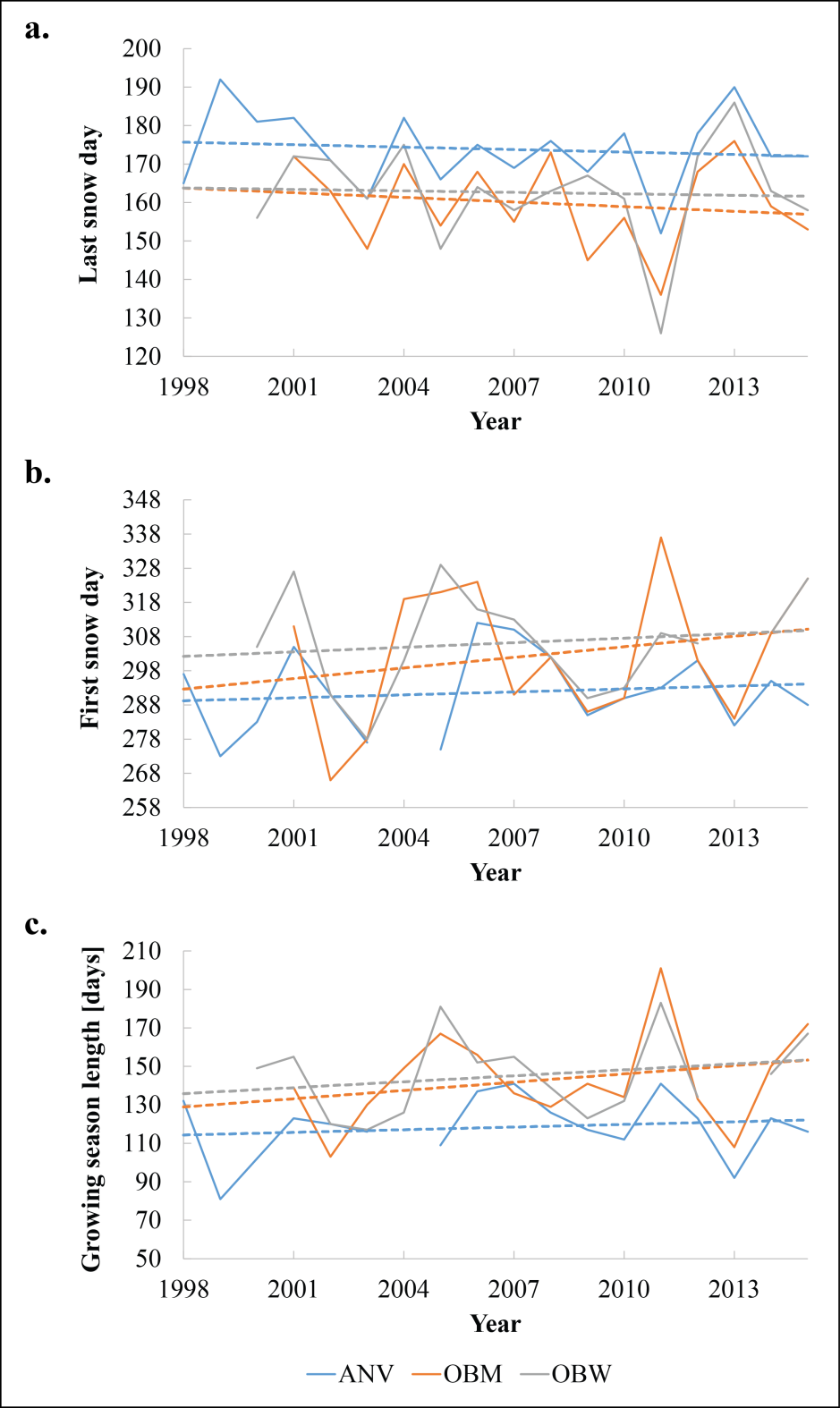
**

**Appendix 7.** Principal Component Analysis based on species composition and cover of the calcareous plant communities: calcareous grasslands (*Seslerion*), windy ridges (*Elynion*) The first axis represents 26.3 % and the second 9.8% of the total variance, respectively. Couples of historical (hollow symbols) and recent (full symbols) records are connected with dotted arrows. Shift of centroids are not represented as they are negligible.


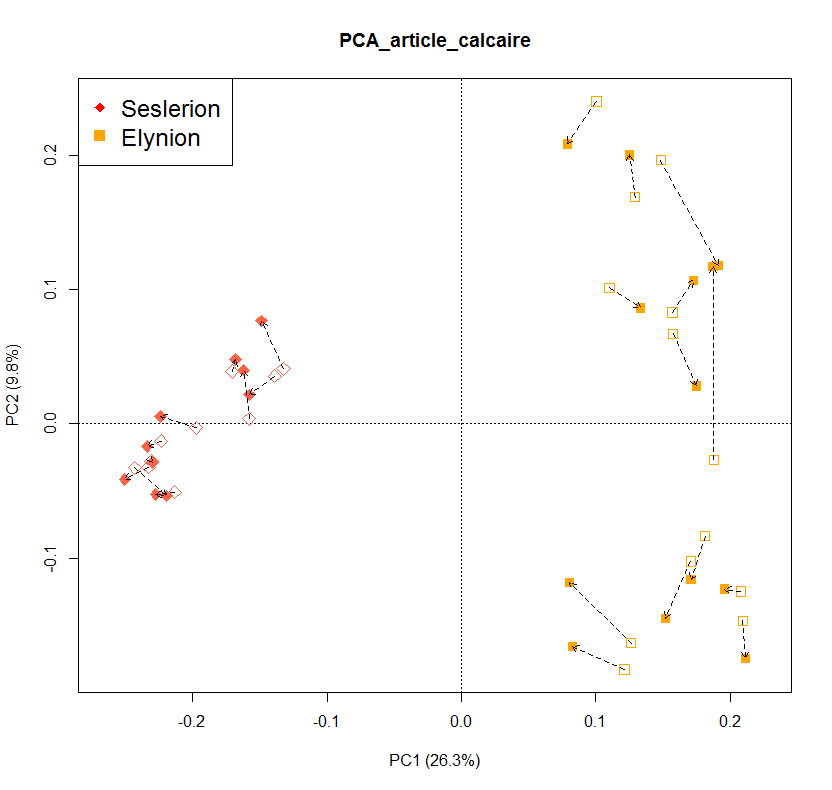


**Appendix 8.** Principal Component Analysis based on species composition and cover of the siliceous plant communities: subalpine siliceous grasslands (*Nardion*), alpine siliceous grasslands (*Caricion curvulae*), typical snowbeds (*Salicion herbaceae*), wet snowbeds (*Caricion bicolori-atrofuscae*). The first axis represents 17.1 % and the second 10.5% of the variance, respectively. Couples of historical (hollow symbols) and recent (full symbols) records are connected with dotted arrows. Shift of centroids are represented by thick arrows, except for *Caricion curvulae* with a negligible shift.


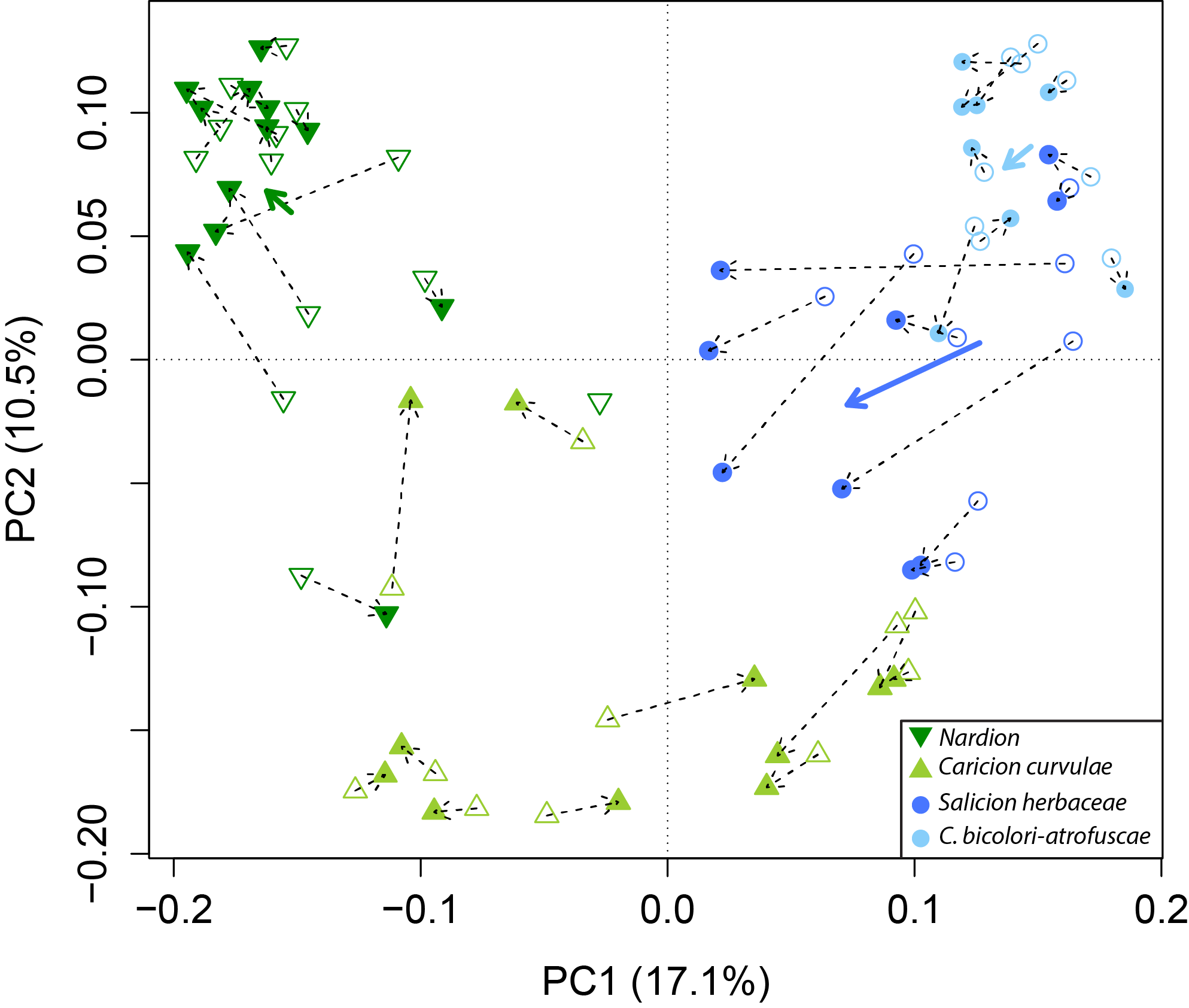


References cited:

Begert M, Schlegel T, Kirchhofer W (2005) Homogeneous Temperature and Precipitation Series of Switzerland from 1864 to 2000. International Journal of Climatology **25**, 65-80.

Delarze R, Gonseth Y (2008) Guide des milieux naturels de Suisse. 2nd ed. Rossolis, Bussigny, 424 pp.
